# Recycling and remanufacturing or technology upgrading? emission reduction decisions of supply chain under carbon cap-and-trade mechanism

**DOI:** 10.1371/journal.pone.0318952

**Published:** 2025-03-31

**Authors:** Xiaoyuan Wu, Wenqing Miao, Xingxian Zhang, Wenjin Zuo, Bingliang Shen

**Affiliations:** 1 School of Foreign Studies, Yiwu Industrial and Commercial College, Yiwu, Zhejiang, China; 2 Digital Intelligence Management Research Institute, Shanghai University of Finance and Economics Zhejiang College, Jinhua, Zhejiang, China; 3 School of Architecture and Engineering, Tongling University, Tongling, Anhui, China; 4 Department of Public Foundations, Shanghai University of Finance and Economics Zhejiang College, Jinhua, Zhejiang, China; West Pomeranian University of Technology, POLAND

## Abstract

Cap-and-trade mechanism can efficiently reduce carbon emissions, which makes it widely used around the world. This paper builds a Stackelberg game model for a manufacturer-led secondary supply chain and analyzes the best pricing, carbon reduction strategies, and the impacts of each variable on equilibrium results when using two emission reduction methods, recycling & remanufacturing, and technology upgrading under the cap-and-trade mechanism. The relationship between the variables was verified through case studies, and the economic benefits and emission reduction effects of the two emission reduction methods were compared. It is found that manufacturers’ profit in the recycling and remanufacturing mode is higher compared to that of the technology upgrading mode when consumer price sensitivity coefficient is low, but its emission reduction effect is less effective; raising the price of carbon trading can effectively increase the carbon reduction per unit low-carbon products and reduce total carbon emissions under the two modes. The government should boost the price of carbon to promote enterprises to reduce emissions, and it can also guide manufacturers to choose the technology upgrading emission reduction mode through other ways to minimize the total carbon emissions.

## 1. Introduction

The total carbon emissions in China have increased dramatically along with becoming the second-fastest growing economy in the world. As the greatest carbon country in the world [1], China contributes 31% of all global carbon outputs, making it a difficult task to achieve the “double carbon” target. Carbon trading policy is an efficient way of encouraging energy conservation in businesses and controlling carbon outputs of the nation [2,3]. Chinese carbon trading system was built late, with carbon trading piloted in some cities only [4]. Chinese carbon trading market was formally started on July 2021, and it becomes the biggest carbon trading market in the world immediately [5]. The listing of Chinese carbon trading market marks a new stage for China to solve environmental problems in a “market-based” way and is also a reflection of China’s active participation in building an international carbon pricing system. Through the carbon trading system, capital inflows into the low-carbon fields [6], stimulating businesses to reduce emissions and save energy, upgrade technology [7], and effectively promote industrial restructuring, ultimately achieving the objectives of reaching carbon peak and becoming carbon neutral in China.

China has included 2,225 firms in the power supply sector in the first execution period of the Chinese carbon market and allocated a certain amount of carbon quotas to each enterprise. This is the first time that China has pressed the responsibility of energy saving and emission reduction to specific enterprises from a macro perspective [8,9], establishing the status of enterprises as the main body of cutting emissions. Three basic ways are available to decrease carbon outputs from the perspective of enterprises. The first is reducing production directly while the carbon emission for each item is unchanged. By reducing carbon output, the overall outputs will decrease [10]. But this way will reduce both the market share and income of enterprises, and it is unfavorable to the long-term growth of enterprises. Most firms will not adopt this way. The second is recycling and remanufacturing, which recycles market-used products and recovers useful parts and components through dismantling and other technologies for remanufacturing [11], and due to the secondary processing based on used parts and components, the unit carbon emission of reproduction will be lower than new products, so enterprises’ total carbon outputs will decrease [12]. The third way is technological upgrading. Through technological upgrading, enterprises develop low-carbon processes and goods, so the emission for each product decreases [13,14]. Enterprises with advanced production technology and low carbon emissions will gain advantages for long-term market competition.

The supply chain has been the “hardest hit area” of carbon emission in China, and emission reduction and energy efficiency should be the goal of all member enterprises in supply chain as well as profit [15]. At present, there are many studies on energy conservation and emission reduction of supply chain, but most of them discuss these topics from the perspective of consumer surplus or social surplus [16–19], and focus more on the effect of recycling and remanufacturing on supply chain [20,21], while fewer on technological innovation and upgrading. In this paper, we simultaneously study two methods of energy conservation and emission reduction based on carbon trading mechanism, recycling and technological upgrading, and compare the energy conservation and emission reduction effects of these two methods and the impacts on member enterprises.

## 2. Literature review

This paper reviews the existing literature on recycling remanufacturing and technology upgrading under cap-and-trade mechanism.

### 2.1. Recycling and remanufacturing under the cap-and-trade mechanism

Carbon trading policy can promote remanufactured products to achieve carbon emission management and reduce the pollution of waste products to the environment, which has been widely used in various industries [22,23]. Remanufacturing under the carbon trading mechanism can utilize the surplus value of old products, reduce carbon emissions and promote the harmonious development of the economy and the environment [24] Recycling and remanufacturing based on cap-and-trade mechanism mainly discusses the case in which the manufacturers were taken as the main body of recycling and remanufacturing [25]. For example, Wei studied two competing supply chains in a duopoly market, one supply chain manufactures new items while the other just recycles and reconstructs, and established five production/remanufacturing game models to identify the best pricing and remanufacturing choices [26] Xia et al. [27,28] analyzed the impact of three remanufacturing emission reduction models on carbon emissions under the carbon trading mechanism, and found that cooperation between OEMs and remanufacturers to reduce emissions can achieve a Nash equilibrium that maximizes supply chain profits and minimizes carbon emissions, and that investment in emission reduction is a key indicator, which affects the cost of outsourcing, price, profit, and level of emission reduction. Guo et al. [29] analyzed the impact of manufacturer remanufacturing and outsourced remanufacturing on recycling and remanufacturing under decentralized and centralized decision-making, and devised a contractual mechanism combining costs, revenues and outsourcing fees to achieve supply chain coordination. Wu [30] studied the producer’s pricing decision when both new products and refurbished goods are produced and consumers’ willingness to pay is consistent with a uniform distribution. Zhang [31] presented the production decisions of an oligopolistic manufacturer that can simultaneously produce new, remanufactured, and refurbished products under carbon trading mechanism considering product life cycle of different purchase areas with the emission savings from various decisions. Wang et al. [32] analyzed the optimal production decisions of capital-constrained manufacturers in supply chain and direct sales models, respectively, and concluded that carbon permit buybacks promote the production of low-carbon reproductions and that lower carbon allowances are more likely to reduce carbon emissions. Bai [33] studied the optimal recycling and reproduction strategies in a reproducing system based on a newsboy model with stable demand distribution and analyzed the effect of government application of cap-and-trade policies. Zhang et al. [34] compared the roles played by different recyclers in reverse battery recycling, where the competition degree among recyclers, the price of carbon trading, and the environmental awareness of consumers all affect the number of recycled batteries. Seyyed-Mahdi [35] analyzed the ideal sustainability grade and pricing determination of a reversed supply chain system with online demand disrupted under carbon trading mechanism. Zhang [36] analysed the impact of authorization fees and government subsidies on remanufacturing and carbon emissions in the case of authorized remanufacturing, where the speed of authorization fee adjustment affects the stability of the system. Sun et al. [37] concluded that consumers’ educational level affects sales of remanufactured products and that government subsidy can incentivize OEMs to adopt remanufacturing decisions. Chai et al. [38] analyzed the optimal decision of a monopoly producer who conducts producing and reproducing in both general and green markets under carbon trading mechanism and found that this policy is valuable for remanufacturing, and carbon price has a great impact on profits and total carbon outputs.

Phased production of remanufactured products also appears in the existing literature. For example, Chang [39] proposed a profit maximization model for monopolistic manufacturers with two-stage production in the demand interchangeable market and the demand distinct market in carbon trading situation and discovered that carbon price could reduce carbon emissions more efficiently compared to carbon allowance, and cap-and-trade mechanism induced manufacturers to upgrade reproducing processes in the substitutable market. Zhu [40] analyzed the optimal decision of oligopolistic manufacturers who produce new items in the initial phase then produce both reproductions and new products in the second phase under carbon trading mechanism, and the effect of this mechanism on production and remanufacturing. Manufacturers’ recycling and remanufacturing, the impact of different sales channels, different intentions to purchase, and different quality of re-products and new items on recycling and remanufacturing were also studied. For example, Yang [41] studied the supply chain remanufacturing and promotion of manufacturers selling products through their channels or retail channels under carbon trading mechanism, then compared the emission reduction effect and profit of supply chain in these two modes. Gan [42] studied the pricing method of reproduction considering different willingness to pay under carbon trading mechanism and proposed a revenue-sharing model to balance the profits of retailers and manufacturers. Turki et al. [43] considered new products as higher quality products and remanufactured products as lower quality products and studied the effects of factors like return rate of old items, machine breakdown, carbon price, and carbon limit on storage according to the differences between the quality of old and new products.

A small amount of literatures studied the effect of various recycling agents on energy saving and outputs reduction in closed-loop supply chains. For example, Fan [44] constructed a Stackelberg game model for a closed-loop supply chain using four different recycling agents then discovered that supply chain members’ profits directly affect the recycling and remanufacturing ratio, and a multi-recycling agent model consisting of online and offline recyclers, manufacturers, and retailers is the best recycling mode, and the carbon trading mechanism can improve the profits of supply chains and manufacturers under this model. Yang [45] built a closed-loop supply chain in which products recycled by producers, retailers, and recyclers under carbon trading mechanism, compared the level of emission reduction and all parties’ profits under three recycling modes. It is found that more carbon emissions would be generated with the lower price of carbon. Niu [46] analyzed environmental risks faced by OEMs and third-party remanufacturers in an authorized reproducing supply chain and the impact of reproducing on environment through big data. Bazan et al. [47] compared the total inventory cost and carbon outputs under classical model and the coordination model in a two-stage supply chain where both retailers and producers have remanufacturing capacity, and found that the coordination model was more economical but not necessarily more environmentally friendly

In summary, recycling and remanufacturing can decrease the emission level of enterprises and improve supply chain members’ revenues in most cases.

### 2.2. Technological upgrading under cap-and-trade mechanism

Cap-and-trade mechanism can incentivize technological innovation and upgrading [48]. The carbon trading mechanism can play the role of the market, promote green technological innovation and upgrading of enterprises, and promote high-quality and green development of cities [49,50]. Manufacturers upgrade existing production technology to cut carbon emissions under cap-and-trade policy [51]. Zhang et al. [52] believed that carbon trading is favorable to reducing carbon emissions. Wang et al. [53] discussed the role of technological upgrading in the reconstruction of global value chains and carbon emission reduction in developing countries such as Russia, Brazil, India, and China, and found that the emission reduction effect in China and India is stronger than that in Russia and Brazil. Han et al. [54] believed that the carbon trading mechanism can produce significant green innovation effects in eastern and western China and cities with high administrative levels, but the effect is weaker in other cities. Zou et al. [55] investigated the competition among members in a low-carbon supply chain under retailers’ inventory constraints, and got the risk aversion characteristics and emission reduction strategies of each member. Pingkuo and Jiahao [56] studied the tripartite evolutionary game among the government, thermal power enterprises and consumers for CCUS technology upgrading, and found that the investment cost of CCUS technology is an important factor for thermal power enterprises to invest in this technology, which ultimately achieves the ideal goal of enterprise investment, government incentives and public participation. Ma et al. [57] established a carbon emission reduction alliance game model for the new energy vehicle supply chain, through which the alliance can incentivize carbon emission reduction and increase the profit of each participant. Kang and Tan [58] analyzed the investment decisions of suppliers and manufacturers in carbon abatement technologies and found that the revenue coefficient determines whether corporation free-rider or not. Based on digital technology and green technology, Kang et al. [59] proposed applying blockchain technology to supply chain carbon emission reduction to build a bilateral synergy mechanism to promote the sales of low-carbon products.

To increase the manufacturers’ incentive to upgrade their technology, contracts are made between retailers and manufacturers to enhance the greening of whole supply chain [60]. For example, Zhi et al. [61] investigated a two-stage supply chain, in which manufacturers’ cost-sharing contract was the core and followed by retailers’ and discovered that suppliers’ technology level of carbon emission reduction affects the contract implementation. Guo et al. [62] discussed the role of three emission reduction planes for a two-stage humanitarian supply chain then discovered that consumer carbon sensitivity coefficients, emission reduction goals, and carbon prices would affect supply chain emission reduction strategies, and price discounting agreements could enable supply chain coordination. Mondal and Giri [63] constructed four decision-making models for a two-stage sustainable supply chain with environmentally conscious retailers and manufacturers in the cap-and-trade mechanism, and found that the revenue-sharing model could lower the carbon outputs and improve the revenues of manufacturers and retailers, and higher carbon price could help boost the green degree of items. Fang and Ma [64] proposed a technology optimization model under heterogeneous agents and non-deterministic carbon trading prices and analyzed the effects of market share, sellers’ discount factors, and carbon prices on carbon emission reduction and new technology adoption, and discovered that cap-and-trade mechanism can successfully lower carbon outputs and promote the adoption of new technologies by manufacturers.

A small amount of literatures have also analyzed the effects of substandard rates, distribution channels, inventory, carbon price stability, and product characteristics on supply chain innovation and carbon emission reduction [65,66]. For example, Qu [67] studied the optimization energy saving and emission reduction model of a two-stage closed supply chain with a substandard rate under carbon trading mechanism, and supply chain is impacted by substandard rate, carbon trading price, and other factors causing the imbalance of cost and emission. Yang et al. [68] analyzed reduction input and supply chains’ effect of different channels under the carbon trading mechanism. Jiang et al. [3] analyzed the effect of emissions ceiling and other relevant factors on the strategies of all parties, total costs, and emissions in a green VMI mode with the introduction of cap-and-trade mechanism, and discovered that cap-and-trade policy would raise the cost of supply chain to some extent. Xu et al. [69] analyzed the optimal pricing and production decisions for a supply chain that manufactures substitutes or complementary goods simultaneously on a per-order basis under carbon trading mechanism, but this mechanism might not always be effective in lowering emissions. Ren and Zhou [70] constructed an unpredictable mixed-integer supply chain LP model considering full life-cycle emissions for PVC products using carbon trading mechanism with market uncertainty and concluded that market uncertainty affected the carbon-intensive production sectors.

In summary, carbon trading mechanism can work in most cases, forcing enterprises to upgrade their technology and green transformation from the perspective of national policies, and promoting them to carry out low-carbon development. However, the above two types of literatures only study the supply chain emission reduction from one aspect of remanufacturing or technology upgrading. But which emission reduction method works better? No literature analyzes remanufacturing and technology upgrading at the same time. Based on the cap-and-trade mechanism, this paper will analyze both cases of manufacturers producing common products and recycled products or only low-carbon goods, then compare the impacts of remanufacturing and technology upgrading on production planning, emission reduction effects, pricing, and economic benefits. Based on consumer heterogeneity and considered the different consumers of remanufactured products and new products, this paper constructs a dual-product carbon emission reduction model in supply chain in which manufacturers sell new and remanufactured products to two separate markets. It extends the research on manufacturers’ independent emission reduction through multiple methods.

## 3. Model assumptions

Due to personal characteristic, preference, income level, genetic makeup, educational level, external environment, and information asymmetry, consumers’ behavior and choices exhibit diversity and differentiation [71,72]. Based on the assumption in literature [73] that consumers have a preference for low carbon, this paper assumes that consumers are sensitive to prices of products and carbon emissions. In order to reflect the difference from other articles, basing on consumer heterogeneity, considering that consumers of remanufactured products and new products belong to different audiences, the product is launched through precise market segmentation, and independent demand functions for new products and remanufactured products are constructed. The specific parameters are shown in Table 1.

**Table 1:** Parameter Description Table.

parameters		decision variables	
$c_{1}$	production cost per unit of new products	$q_{1}$	sales of common products
$c_{2}$	production cost per unit of reproductions	$q_{2}$	sales of reproductions
$c_{3}$	production cost per unit of low-carbon products	$q_{3}$	sales of low-carbon products
*α*	market potential	$w_{1}$	wholesale price of common products
*β*	consumer price sensitivity coefficient	$w_{2}$	wholesale price of reproductions
*λ*	consumer emission reduction sensitivity coefficient	$w_{3}$	wholesale price of low-carbon products
*θ*	carbon emission discount factor of remanufactured products	$p_{1}$	retail price of common products
*ε*	abatement cost coefficient	$p_{2}$	retail price of reproductions
$e_{1}$	carbon emission per unit of common product	$p_{3}$	retail price of low-carbon products
$p_{c}$	carbon price	*e*	carbon emission reduction per unit of low-carbon product

Assumption 1: A leading manufacturer and a following retailer compose a supply chain. The total carbon emissions of the supply chain are generated in the production process by manufacturer. The product transportation and sales processes do not generate carbon emissions, so as to facilitate the comparison of the effects of these two emission reduction methods.

Assumption 2: The carbon emission per unit of common product is $e_{1}$. Since remanufactured products are remanufactured based on recycled products, their carbon emission is less than common products. The carbon emission discount factor of remanufactured products is *θ* (0<θ<1), then the carbon emission per unit of re-products is $\theta e_{1}$, so each re-products’ emission reduction is $e_{1}-\theta e_{1}$. Enterprises can produce low-carbon products through technological upgrading. Compared with common products, low-carbon goods have lower outputs, and the carbon emission reduction per unit of low-carbon products is *e*. Due to the limitation of existing technology, zero emission cannot be fully realized, so $0<e<e_{1}$. Then each low-carbon product’s carbon emission is $e_{1}-e$.

Assumption 3: The government gives the manufacturer a free carbon allocation *E*. If the carbon emissions of manufacturer exceed this carbon quota, the excess amount should be purchased from the carbon market. On the other hand, when the manufacturers’ outputs do not reach this carbon quota, they can sell the excess amount in the carbon market. Carbon trading price is $p_{c}$.

Assumption 4: The market demand for common product without carbon emission reduction is q=α−βp. The market potential is *α* and the consumer price sensitivity coefficient is β. Consumers favor low carbon and *λ* is the consumer emission reduction sensitivity coefficient. The market demand for re-products and low-carbon items with different degrees of carbon reduction is q=α−βp+λe. The value of the market potential does not affect the analysis, so let α=1 for the convenience of calculation.

Assumption 5: The cost of enterprise technological upgrading is a concave function of *e*. Assume that the cost of technological upgrading is $\frac{1}{2}\varepsilon e^{2}$, where *ε* is the abatement cost coefficient (ε>0).

Assumption 6: Unit product$c_{2}$ion cost of new product is $c_{1}$, and the re-product’s is . Because the re-product is reprocessed on the basis of recycled product, its cost is less than new item, so $c_{2}<c_{1}$. The prices of common product, re-product and low carbon product are $p_{1}$, $p_{2}$, $p_{3}$, while the wholesale price of these three products are $w_{1}$, $w_{2}$, $w_{3}$.

## 4. Model solving and analyzing

### 4.1. Recycling and remanufacturing strategy

The manufacturer produces the ordinary product and remanufactures the recycled product simultaneously. By remanufacturing the recycled product, the quality requirements of the new product can be fully achieved, and the reproduction cost is cheaper than that of new product. The common items and the recycled reproduced items are sold to two markets respectively. The remanufactured product is processed and remanufactured based on the recycled product, so its carbon emission is less than common product’s as $\theta e_{1}$. Common items and the low-carbon items are wholesaled to the retailer with a wholesale price of $w_{1}$ and $w_{2}$ respectively by the manufacturer, and the retailer sell them at $p_{1}$ and $p_{2}$ respectively.

The demand for common products is $q_{1}=1-\beta p_{1}$ Each reproductions’ carbon reduction is $\left(1-\theta \right)e_{1}$ compared with common products, then the market demand for re-products is $q_{2}=1-\beta p_{2}+\lambda \left(1-\theta \right)e_{1}$

The manufacturer’s profit is $\pi_{m}^{1}=\left(w_{1}-c_{1}\right)q_{1}+\left(w_{2}-c_{2}\right)q_{2}+p_{c}\left[E-e_{1}q_{1}-\theta e_{1}q_{2}\right]$ (1)

In equation (1), $\left(w_{1}-c_{1}\right)q_{1}$ is the manufacturer’s revenue of producing and selling common products, $\left(w_{2}-c_{2}\right)q_{2}$ is that of remanufactured products, $p_{c}\left[E-e_{1}q_{1}-\theta e_{1}q_{2}\right]$ is the manufacturer’s revenue or cost from selling or purchasing carbon allowances.

The retailer sells ordinary products and reproductions with a profit of $\pi_{r}^{1}=\left(p_{1}-w_{1}\right)q_{1}+\left(p_{2}-w_{2}\right)q_{2}$ (2)

Theorem 1: The optimal wholesale price, retail price and sales volume in the recycling and remanufacturing case is $\left(\frac{\text{1}+c_{1}\beta +p_{c}e_{1}\beta }{2\beta },\frac{1-\lambda \left(1-\theta \right)e_{1}+c_{2}\beta +p_{c}\theta e_{1}\beta }{2\beta }\right)$, $\left(\frac{3+c_{1}\beta +p_{c}e_{1}\beta }{4\beta },\frac{3+\lambda \left(1-\theta \right)e_{1}+\beta c_{2}+p_{c}\theta e_{1}\beta }{4\beta }\right)$, $\frac{1-c_{1}\beta -p_{c}e_{1}\beta }{4}$, $\frac{1+3\lambda \left(1-\theta \right)e_{1}-\beta c_{2}-p_{c}\theta e_{1}\beta }{4}$.

Proof: For this supply chain is manufacturer-driven, we adopt the inverse generation method to find the optimal solution of this problem. Hesse matrix that corresponds to the profit formula of the retailer is $\text{H}\left(\pi_{r}^{1}\right)=\left|\begin{array}{cc} -2\beta & 0\\ 0 & -2\beta \end{array}\right|$, −2β<0 and $4\beta^{2}>0$. This matrix is negative definite and has a maximum result. Calculate the partial derivatives of $p_{1}$ and $p_{2}$ for formula (2), thus we can obtain:


$$\frac{\partial \pi_{r}^{1}}{\partial p_{1}}=1-2\beta p_{1}+w_{1}\beta$$

$$\frac{\partial \pi_{r}^{1}}{\partial p_{2}}=1-2\beta p_{2}+\lambda \left(1-\theta \right)e_{1}+w_{2}\beta$$

Let the above formula be set to 0, and the concurrent formulas are solved as


$$\{\begin{array}{c} p_{1}=\frac{1+w_{1}\beta }{2\beta }\\ p_{2}=\frac{1+\lambda \left(1-\theta \right)e_{1}+w_{2}\beta }{2\beta } \end{array}$$ (3)

Substituting the retail price into equation (1), profit formula of manufacturer is got, and its corresponding Hesse matrix is $\text{H}\left(\pi_{m}^{1}\right)=\left|\begin{array}{cc} -\beta & 0\\ 0 & -\beta \end{array}\right|$, which is negative definite due to −β<0 and $\beta^{2}>0$. Therefore, the manufacturer’s profit formula has maximum value.

Calculate the partial derivative of $w_{1}$, $w_{2}$ for the profit function of manufacturer, then let it equal to 0; thus, we can find:


$$\frac{\partial \pi_{m}^{1}}{\partial w_{1}}=\frac{1}{2}-\beta w_{1}+\frac{c_{1}\beta }{2}+\frac{p_{c}e_{1}\beta }{2}=0$$

$$\frac{\partial \pi_{m}^{1}}{\partial w_{2}}=\frac{1}{2}-\frac{\lambda 1-\text{θ}e_{1}}{2}-\beta w_{2}+\frac{\beta c_{2}}{2}+\frac{p_{c}\theta e_{1}\beta }{2}=0$$

The wholesale prices of the two products obtaimned are $w_{1}=\frac{1+c_{1}\beta +p_{c}e_{1}\beta }{2\beta }$, $w_{2}=\frac{1-\lambda \left(1-\theta \right)e_{1}+c_{2}\beta +p_{c}\theta e_{1}\beta }{2\beta }$.

Substituting the above wholesale prices into formula (3), we obtain the retail prices of common product and re-product as $p_{1}=\frac{3+c_{1}\beta +p_{c}e_{1}\beta }{4\beta }$, $p_{2}=\frac{3+\lambda \left(1-\theta \right)e_{1}+\beta c_{2}+p_{c}\theta e_{1}\beta }{4\beta }$.

Accordingly, the price margin between common product and the remanufactured product is $p_{1}-p_{2}=\frac{\beta \left(c_{1}-c_{2}\right)-\lambda \left(1-\theta \right)e_{1}+p_{c}e_{1}\beta \left(1-\theta \right)}{4\beta }$, and the sale volume of common product and remanufactured product are $q_{1}=\frac{1-c_{1}\beta -p_{c}e_{1}\beta }{4}\frac{\partial q_{2}}{\partial \lambda }>0$, $q_{2}=\frac{1+3\lambda \left(1-\theta \right)e_{1}-\beta c_{2}-p_{c}\theta e_{1}\beta }{4}$.

Inference 1: In the mode of recycling and remanufacturing, $\frac{\partial p_{1}}{\partial \beta }=<0$, $\frac{\partial p_{1}}{\partial p_{c}}>0$, $\frac{\partial p_{2}}{\partial \beta }=<0$, $\frac{\partial p_{2}}{\partial \lambda }>0$, $\frac{\partial p_{2}}{\partial p_{c}}>0$, $\frac{\partial p_{2}}{\partial \theta }<0$, $\frac{\partial q_{1}}{\partial \beta }<0$, $\frac{\partial q_{1}}{\partial p_{c}}<0$, $\frac{\partial q_{2}}{\partial \beta }<0$, $\frac{\partial q_{2}}{\partial \theta }<0$, $\frac{\partial q_{2}}{\partial p_{c}}<0$.

Proof:

$\frac{\partial p_{1}}{\partial \beta }=\frac{4c_{1}\beta +4p_{c}e_{1}\beta -4\left(3+c_{1}\beta +p_{c}e_{1}\beta \right)}{16\beta^{2}}=-\frac{3}{4\beta^{2}}<0$, $\frac{\partial p_{2}}{\partial \beta }=\frac{4\beta \left(c_{2}+p_{c}\theta e_{1}\right)-4\left[3+\lambda \left(1-\theta \right)e_{1}+\beta c_{2}+p_{c}\theta e_{1}\beta \right]}{16\beta^{2}}=-\frac{12+4\lambda \left(1-\theta \right)e_{1}}{4\beta^{2}}<0$. The retail price of both goods and the consumer price sensitivity coefficient are inversely connected.

$\frac{\partial q_{1}}{\partial \beta }=-\frac{c_{1}+p_{c}e_{1}}{4}<0$, $\frac{\partial q_{2}}{\partial \beta }=-\frac{c_{2}+p_{c}\theta e_{1}}{4}<0$. The sales of both products are negatively correlated with the consumer price sensitivity coefficient.

$\frac{\partial p_{2}}{\partial \lambda }=\frac{\left(1-\theta \right)e_{1}}{4\beta }$, because 0<θ<1, $\frac{\partial p_{2}}{\partial \lambda }>0$. $\frac{\partial q_{2}}{\partial \lambda }=\frac{3\left(1-\theta \right)e_{1}}{4}>0$, the retail price and sales volume of re-products are both connected favorably with the consumer low-carbon sensitivity coefficient.

$\frac{\partial p_{2}}{\partial \theta }=-\frac{\lambda e_{1}}{4\beta }<0$, $\frac{\partial q_{2}}{\partial \theta }=-\frac{3\lambda e_{1}+p_{c}e_{1}\beta }{4}<0$. Both the retail price and sales volume of remanufactured products are inversely associated with the remanufactured carbon discount coefficient.

$\frac{\partial p_{1}}{\partial p_{c}}=\frac{e_{1}}{4}>0$, $\frac{\partial p_{2}}{\partial p_{c}}=\frac{\theta e_{1}}{4}>0$, both products’ retail prices have a positive correlation with carbon trading prices; $\frac{\partial q_{1}}{\partial p_{c}}=-\frac{e_{1}\beta }{4}<0$, $\frac{\partial q_{2}}{\partial p_{c}}=-\frac{\theta e_{1}\beta }{4}<0$, the sales of both products are negatively correlated with them.

Inference 1 demonstrates that in the case of recycling and remanufacturing, a large consumer price sensitivity coefficient will result in a lower acceptance of high prices and a higher acceptance of low prices by consumers, so the product’s retail price get lower. At the same time, customers who are more sensitive to price will be less accepting of current price in the case of an unchanged price, and less likely to induce purchase behavior, which will lead to less market demand. The higher the consumer low-carbon sensitivity coefficient, the stronger the willingness of consumers to buy remanufactured goods, the stronger the acceptance of re-product price will be, and the higher the demand and price of re-product will be, when the emission reduction per re-product is fixed. A larger emission discount coefficient of remanufactured goods will lead to a smaller emission reduction compared with common products. Because consumers prefer low carbon, the purchase willingness of remanufactured goods will decrease, and the price of re-products will decrease. With other conditions unchanged, along with the rising of carbon price, the costs of carbon trading for enterprises increase, which need to be apportioned to each product, make both products’ retail price rise. Consumers are price sensitive, leading to a decrease in market demand.

In recycling and reproducing mode, manufacturer’s overall emission is $E_{1}=\frac{e_{1}\left(1-c_{1}\beta -p_{c}e_{1}\beta \right)}{4}+\frac{\theta e_{1}\left[1+3\lambda \left(1-\theta \right)e_{1}-\beta c_{2}-p_{c}\theta e_{1}\beta \right]}{4}$.

Inference 2: The overall carbon emission of recycling and reproducing mode is inversely associated with the price sensitivity coefficient and carbon trading price.

Proof: $\frac{\partial E_{1}}{\partial \beta }=-\frac{e_{1}c_{1}+p_{c}e_{1}^{2}}{4}-\frac{c_{2}\theta e_{1}+p_{c}\theta^{2}e_{1}^{2}}{4}<0$, so there is a negative correlation between overall carbon emission and *β*. The overall carbon emission will get lower when the price sensitivity coefficient gets higher. If consumer’s price sensitivity get higher, the willingness to purchase get weaker at the same price, the sales of both common and low carbon items fall down, and the overall emission of manufacturers decreases too.

$\frac{\partial E_{1}}{\partial \lambda }=\frac{3\theta \left(1-\theta \right)e_{1}^{2}}{4}>0$, $E_{1}$ and *λ* are positively correlated. That is, the more sensitive consumers are to carbon emission reduction, the smaller the carbon emissions under the recycling and remanufacturing mode.

$\frac{\partial E_{1}}{\partial p_{c}}=-\frac{e_{1}^{2}\beta +\theta^{2}e_{1}^{2}\beta }{4}<0$, so the total carbon emission and the carbon price are negatively correlated. The carbon trading cost of manufacturers rises with the rising of carbon price, and manufacturers will maintain their profits through raising price, which makes consumers’ demand lower. Manufacturers produce according to demand, so the production of both common and low-carbons decreases, which makes the total emission of enterprises lower.

### 4.2. Low-carbon emission reduction technology upgrading strategy

Manufacturers reduce the emissions of per product and direct production of low-carbons through technological upgrading. Low-carbon products are also new products. Except for the different production processes, its raw material inputs are the same as common products, so its producing cost equals to the ordinary products. That is, $c_{3}=c_{1}$. The consumer demand of this low-carbon product is $q_{3}=1-\beta p_{3}+\lambda e$.

In the mode of technological upgrading, the manufacturer’s profit is $\pi_{m}^{2}=\left(w_{3}-c_{1}\right)q_{3}-\frac{1}{2}\varepsilon e^{2}+p_{c}\left[E-\left(e_{1}-e\right)q_{3}\right]$. (4)

In equation (4), $\left(w_{3}-c_{1}\right)q_{3}$ is manufacturer’s profit generated by the sale of low-carbon goods, $\frac{1}{2}\varepsilon e^{2}$ is the investment cost of technology upgrading, and $p_{c}\left[E-\left(e_{1}-e\right)q_{3}\right]$ represents the manufacturer’s cost or income from carbon trade.

At this point the retailer’s profit is $\pi_{r}^{2}=\left(p_{3}-w_{3}\right)q_{3}$. (5)

Theorem 2: In the case of technological upgrading, when $\varepsilon >\frac{{\left(\lambda +\beta p_{c}\right)}^{2}}{4\beta }$the optimal carbonreduction, retail price, wholesale price and the sales volume are $\frac{\left(p_{c}\beta +\lambda \right)\left(1-c_{1}\beta -p_{c}e_{1}\beta \right)}{4\beta \varepsilon -{\left(\lambda +\beta p_{c}\right)}^{2}}$, $\frac{\varepsilon \left(3+\beta c_{1}\right)-\lambda^{2}c_{1}-p_{c}\left(p_{c}\beta +p_{c}\beta e_{1}\lambda +\beta c_{1}\lambda +\lambda^{2}e_{1}-\beta e_{1}\varepsilon +\lambda \right)}{4\beta \varepsilon -{\left(\lambda +\beta p_{c}\right)}^{2}}$, $\frac{p_{c}\left[p_{c}\left(\beta +\lambda \beta e_{1}\right)-2\beta e_{1}\varepsilon +\lambda \left(1+c_{1}+\lambda e_{1}\right)\right]+2\varepsilon \left(1+\beta c_{1}\right)-\lambda^{2}c_{1}}{4\beta \varepsilon -{\left(\lambda +\beta p_{c}\right)}^{2}}$, $\frac{\beta \varepsilon \left(1-c_{1}\beta -p_{c}\beta e_{1}\right)}{4\beta \varepsilon -{\left(\lambda +\beta p_{c}\right)}^{2}}$.

Proof: Adopting the inverse proof method, we first compute the retailer profit function with respect to $p_{3}$ and set it 0 to get $\frac{\partial \pi_{r}^{2}}{\partial p_{3}}=1+\lambda e+w_{3}\beta -2\beta p_{3}=0$.

Get $p_{3}=\frac{1+\lambda e+w_{3}\beta }{2\beta }$. (6)

Substituting this into the demand function, we obtain $q_{3}=\frac{1+\lambda e-w_{3}\beta }{2}$. (7)

Substituting into formula (4) to obtain the manufacturer’s profit function


$$\pi_{m}^{2}=\left(w_{3}-c_{1}\right)\frac{1+\lambda e-w_{3}\beta }{2}-\frac{1}{2}\varepsilon e^{2}+p_{c}\left[E-\left(e_{1}-e\right)\frac{1+\lambda e-w_{3}\beta }{2}\right]$$ (8)

Its corresponding Hessian matrix is $H\left(\pi_{m}^{2}\right)=\left|\begin{array}{cc} -\beta & \frac{\lambda -\beta p_{c}}{2}\\ \frac{\lambda -\beta p_{c}}{2} & \lambda p_{c}-\varepsilon \end{array}\right|$, −β<0$4\beta \varepsilon -{\left(\lambda +\beta p_{c}\right)}^{2}>0$When $\varepsilon >\frac{{\left(\lambda +\beta p_{c}\right)}^{2}}{4\beta }$, $-\beta \lambda p_{c}-\varepsilon -{\frac{\lambda -\beta p_{c}}{2}}^{2}=\text{βε}-{\frac{\lambda +\beta p_{c}}{2}}^{2}>0$. The manufacturer’s profit curve has a maximum value because this matrix is negatively definite.

Taking partial derivatives of $w_{3}$ and *e* for formula (8) and making them equal to 0, we get


$$\frac{\partial \pi_{m}^{2}}{\partial w_{3}}=\frac{1+\lambda e}{2}-\beta w_{3}+\frac{c_{1}\beta }{2}=0$$

$\frac{\partial \pi_{m}^{2}}{\partial e}=\frac{\lambda \left(w_{3}-c_{1}\right)}{2}-\varepsilon e+\frac{p_{c}}{2}+\lambda p_{c}e-\frac{p_{c}w_{3}\beta }{2}-\frac{p_{c}e_{1}\lambda }{2}=0$, The solution of simultaneous equations is obtained as


$$w_{3}=\frac{p_{c}\left[p_{c}\left(\beta +\lambda \beta e_{1}\right)-2\beta e_{1}\varepsilon +\lambda \left(1+c_{1}+\lambda e_{1}\right)\right]+2\varepsilon \left(1+\beta c_{1}\right)-\lambda^{2}c_{1}}{4\beta \varepsilon -{\left(\lambda +\beta p_{c}\right)}^{2}}$$

$$e=\frac{\left(p_{c}\beta +\lambda \right)\left(1-c_{1}\beta -p_{c}e_{1}\beta \right)}{4\beta \varepsilon -{\left(\lambda +\beta p_{c}\right)}^{2}}$$

Substituting them into equations (6) and (7), we get


$$q_{3}=\frac{\beta \varepsilon \left(1-c_{1}\beta -p_{c}\beta e_{1}\right)}{4\beta \varepsilon -{\left(\lambda +\beta p_{c}\right)}^{2}}$$

$$p_{3}=\frac{\varepsilon \left(3+\beta c_{1}\right)-\lambda^{2}c_{1}-p_{c}\left(p_{c}\beta +p_{c}\beta e_{1}\lambda +\beta c_{1}\lambda +\lambda^{2}e_{1}-\beta e_{1}\varepsilon +\lambda \right)}{4\beta \varepsilon -{\left(\lambda +\beta p_{c}\right)}^{2}}$$

In the technology upgrading mode, above optimal solution is substituted to obtain the manufacturer’s overall carbon output $E_{2}=\left[e_{1}-\frac{\left(p_{c}\beta +\lambda \right)\left(1-c_{1}\beta -p_{c}e_{1}\beta \right)}{4\beta \varepsilon -{\left(\lambda +\beta p_{c}\right)}^{2}}\right]\frac{\beta \varepsilon \left(1-c_{1}\beta -p_{c}\beta e_{1}\right)}{4\beta \varepsilon -{\left(\lambda +\beta p_{c}\right)}^{2}}$.

Inference 3: In the technologypgrading mode, the optimal *e* is negatively associated with *ε*, positively associated with the emission sensitivity coefficient *λ*, and positively associated with the carbon price.

Proof: $\frac{\partial e}{\partial \varepsilon }=\frac{\beta \varepsilon \left(1-c_{1}\beta -p_{c}\beta e_{1}\right)\left(-4\beta \right)}{{\left[4\beta \varepsilon -{\left(\lambda +\beta p_{c}\right)}^{2}\right]}^{2}}$, for $\beta \varepsilon \left(1-c_{1}\beta -p_{c}\beta e_{1}\right)>0$, The optimal *e* is negatively connected to *ε*.

$\frac{\partial e}{\partial \lambda }=\frac{1-c_{1}\beta -p_{c}e_{1}\beta +\left(p_{c}\beta +\lambda \right)\left(1-c_{1}\beta -p_{c}e_{1}\beta \right)\left(2\lambda +2\beta p_{c}\right)}{{\left[4\beta \varepsilon -{\left(\lambda +\beta p_{c}\right)}^{2}\right]}^{2}}$, for e>0 and $4\beta \varepsilon -{\left(\lambda +\beta p_{c}\right)}^{2}>0$, so $1-c_{1}\beta -p_{c}e_{1}\beta >0$, $p_{c}<\frac{1-c_{1}\beta }{e_{1}\beta }$. So $\frac{\partial e}{\partial \lambda }>0$, then *e* and *λ* are positively correlated.

$\frac{\beta \left[4\beta \varepsilon \left(1-c_{1}\beta -p_{c}e_{1}\beta \right)+\left(1-c_{1}\beta -p_{c}e_{1}\beta \right){\left(\lambda +\beta p_{c}\right)}^{2}-4\beta \varepsilon e_{1}\left(p_{c}\beta +\lambda \right)+e_{1}{\left(p_{c}\beta +\lambda \right)}^{3}\right]}{{\left[4\beta \varepsilon -{\left(\lambda +\beta p_{c}\right)}^{2}\right]}^{2}}>\frac{\beta \left(1-c_{1}\beta -p_{c}e_{1}\beta \right){\left(\lambda +\beta p_{c}\right)}^{2}}{{\left[4\beta \varepsilon -{\left(\lambda +\beta p_{c}\right)}^{2}\right]}^{2}}>0$ $p_{c}$

Inference 3 shows that in the technology upgrading mode the larger *ε* is, the smaller the reduction effect that can be achieved with the same total capital investment in outputs reduction, which means a smaller reduction per product. Demand will rise because consumers have a stronger willingness to purchase large carbon emission reduction products as the carbon emission reduction sensitivity increases. Manufacturers will increase abatement efforts to meet consumers’ demand so that the ideal carbon abatement of each product rises. Additionally, an increase in sales volume increases the manufacturer’s income, and more funds are available for enterprise technology upgrading, which also leads to an increase in the optimal emission reduction per item. It is positively related to the carbon price, which means that the cost of purchasing or selling carbon allowances will get higher along with carbon price rising. Manufacturers usually reduce carbon emissions of each product through energy conservation and output reduction methods for the sake of corporate social responsibility and long-term development, in order to lower enterprises’ carbon emissions and reduce the expense of purchasing carbon rights or improve the income from selling carbon rights, and increase manufacturers’ profits.

## 5. Analysis of calculation example

According to reference [20], the market volume is regarded as 1 unit, and the actual data of the corresponding two emission reduction methods are obtained through field investigation of a manufacturing supply chain in Zhejiang Province, China, which is reduced in equal proportion according to the degree of market volume reduction. Let the production cost per unit of new products and recycled reproductions be 0.6 yuan per unit and 0.3 yuan per unit, ε=2 β=0.4, λ=0.5, θ=0.5, $e_{1}=0.8$ ton per unit, carbon allowance E=0.35 ton, and take $p_{c}=1$ yuan per ton, and use MATLAB software for simulation. The effectiveness of emission reduction and economic benefits of the two emission reduction methods are verified by simulation. The above values are substituted into the equilibrium results in the previous chapter, and the equilibrium results under these two cases are displayed in Table 2. The retail price and wholesale price of the technology upgrading strategy are higher compared to those of recycling and remanufacturing strategy, while the sales volume, manufacturers’ and retailers’ profits, and total carbon emissions are lower than the recycling and remanufacturing strategy.

**Table 2:** Comparison table of equilibrium results in two cases.

	Recycling and remanufacturing		Technology upgrading
	Common new products	Remanufactured products	Low-carbon new products
Wholesale price (yuan)	1.95	1.35	2.13
Retail price (yuan)	2.225	2.175	2.339
Sales volume (unit)	0.11	0.33	0.14728
*e* (ton)	/		0.16569
$\pi_{m}$ (yuan)	0.625		0.454464
(yuan)	$\pi_{r}$0.3025		0.030781
Total carbon emission (ton)	0.22		0.09342117

### 5.1. Effect of *θ* on equilibrium results

**Fig 1:**
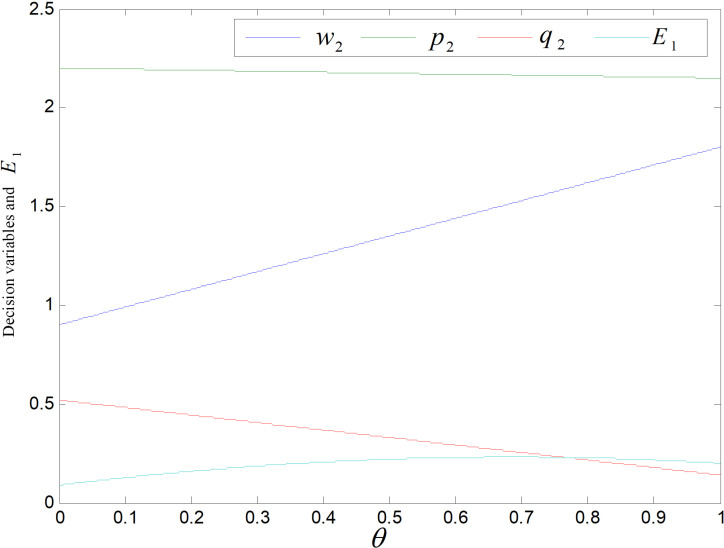
Effect of *θ* on each decision variable and $E_{1}$.

*θ* is the carbon emission discount factor for remanufactured products, which only affects on the decision variable in the recycling and remanufacturing mode, independent of the technology upgrading mode. Fig 1 shows the effect of *θ* on product price and $E_{1}$. The common products’ wholesale and retail prices are $w_{1}=1.95$ and $p_{1}=2.225$, which are not affected by *θ* and are not marked in the figure. Re-products’ wholesale price increases, while the price of retail and demand decrease, and the overall carbon emission decreases after rising. Each re-product’s carbon emission, the carbon allowance occupied, the cost apportioned to each re-product and the wholesale price all rise as the discount coefficient of carbon emission increases. At the same time, per re-product’s carbon emission rises, consumers have low carbon preference, their willingness to purchase re-products decreases, the demand decreases, and retailers will use the strategy of price reduction to increase sales, so the retail price decreases. When *θ* is small, per re-product’ carbon output’s rising is larger than the decrease in demand, and the overall carbon emission rises. When *θ* is higher, each re-product’s carbon emission rising is less than the decrease of demand, so the total carbon emission declines.

**Fig 2:**
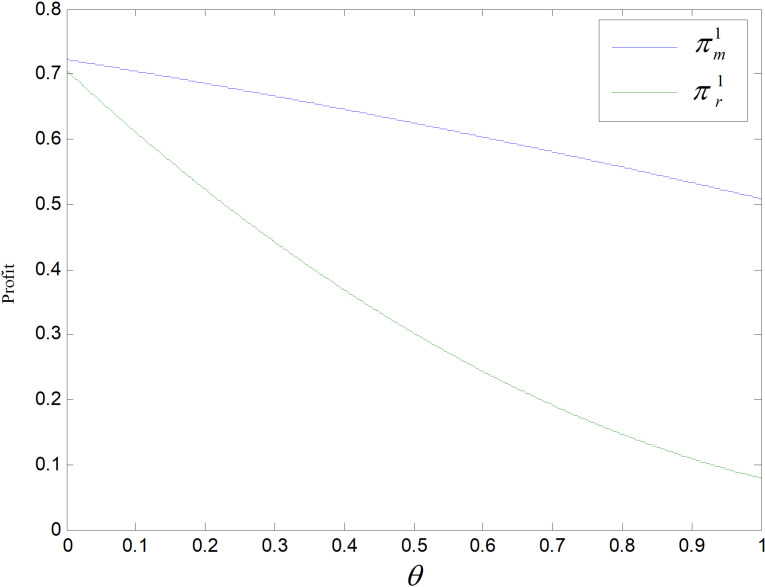
Effect of *θ* on profit.

Fig 2 displays the relationship between manufacturers’ and retailers’ profits and the discount factor of re-products’ carbon emission *θ* in recycling mode. Both manufacturer’s and retailer’s profits decline as *θ* increases, wi*θ*th a larger decrease for the retailer. The larger is, the greater per remanufactured item’s carbon emission, the lower the consumer market demand, and the lower the producer’s incomes. The profit of retailer is generated by the difference between the retail price and the wholesale price. As *θ* increases, the price of wholesale increases, the price of retail decreases, the difference keeps getting smaller, while the demand decreases, so the profit of manufacturer decreases less than the retailer's.

### 5.2. Effect of *ε* on equilibrium results

**Fig 3:**
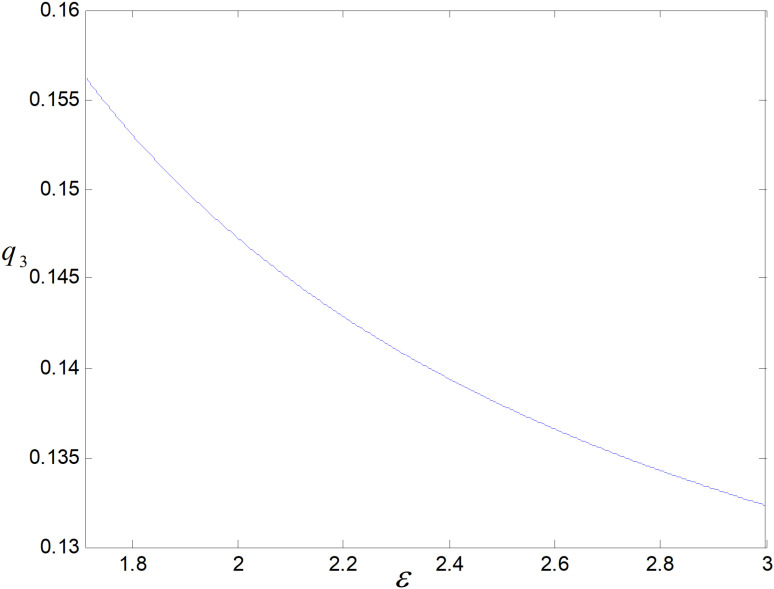
Effect of *ε* on $q_{3}.$.

**Fig 4:**
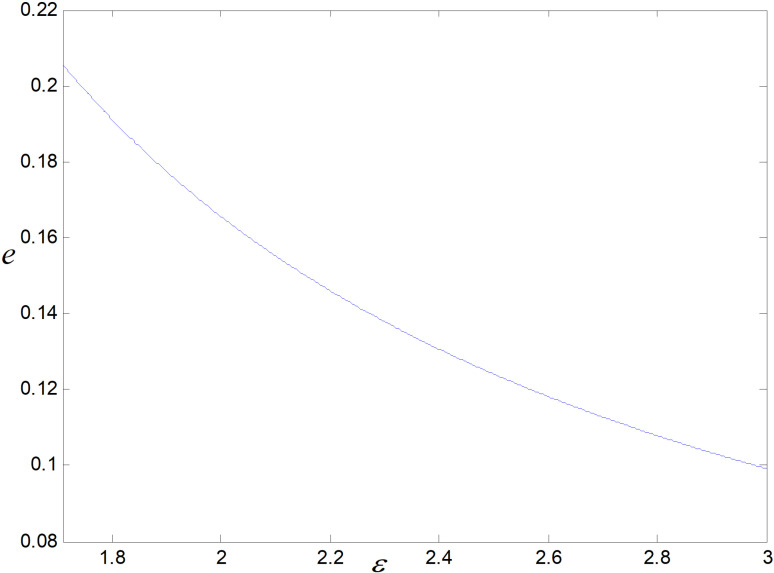
Effect of *ε* on *e.*

**Fig 5:**
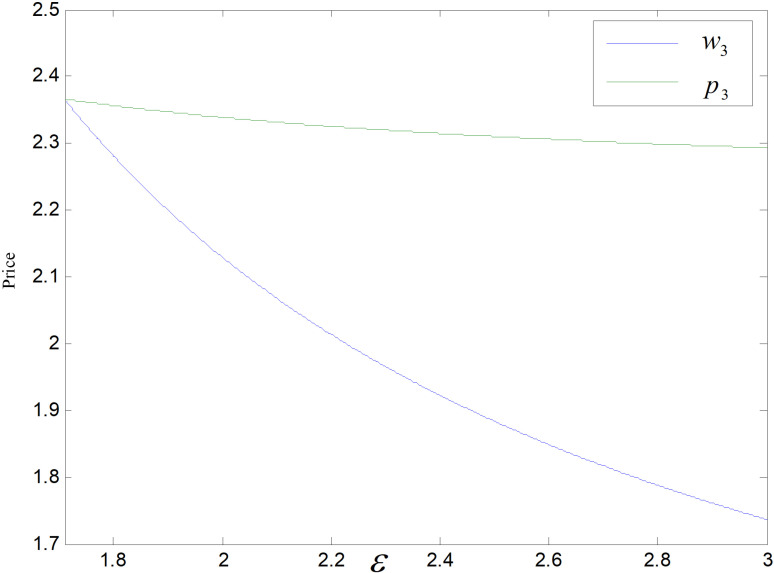
Effect of *ε* on price.

**Fig 6:**
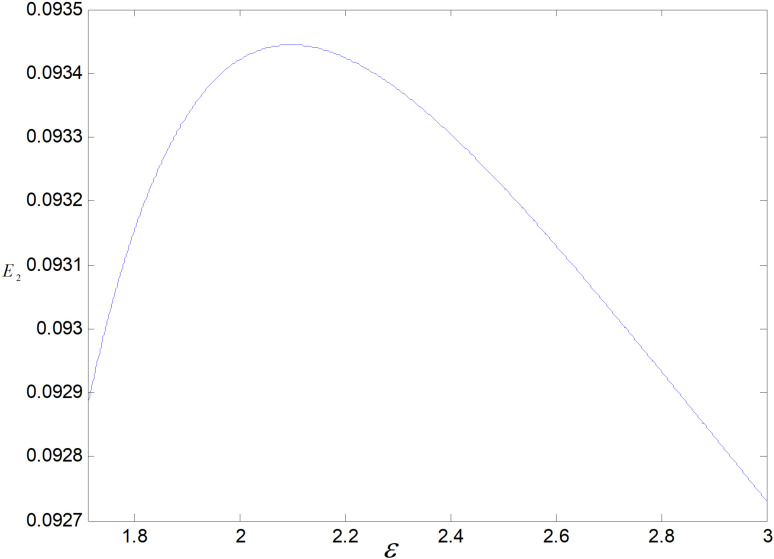
Effect of *ε* on $E_{3}$.

**Fig 7:**
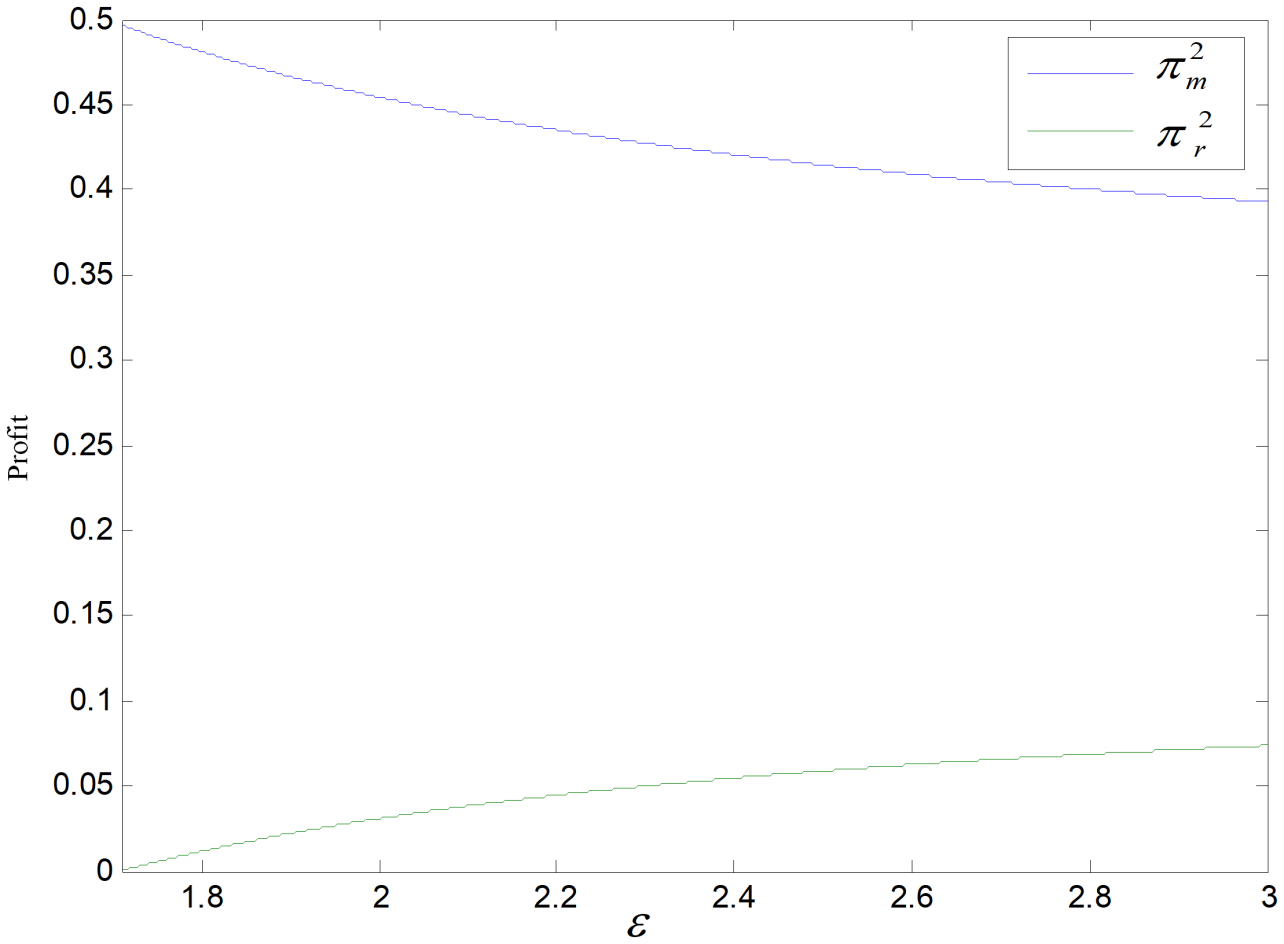
Effect of *ε* on profit.

When other parameters take constant values, we get $\varepsilon \in \left[1.71,3\right]$. *ε* is the cost coefficient of technological upgrading, which does not affect each decision variable in the recycling mode, and its effects on demand, optimal emission reduction factor, price, total carbon emission, and profit in the technological upgrading mode are shown in Figs 3–7.

The effect of *ε* on low-carbon items’ demand is shown in Fig 3. As *ε* rises, the worse the reduction effect achieved under a same technology upgrade cost investment, i.e., per low-carbon item’s optimal emission reduction becomes smaller and smaller, as illustrated in Fig 4. Consumers have low carbon preference. The smaller the emission reduction, the lower the consumer’s willingness to purchase, and the market demand decreases. Consumers are also price sensitive, and to increase consumer purchase demand, companies try to increase consumer demand for their products by reducing prices. Manufacturers do not directly contact consumers. To promote retailers to increase product sales, they will be given a more substantial reduction in wholesale prices, as shown in Fig 5.

Fig 6 shows the effect of on total carbon emissions under technology upgrading mode. As *ε* increases, total carbon emissio*ε*ns rise and then fall. *ε* increases, per low-carbon product’s carbon emission rises, and demand falls. When *ε* is small, decrease in demand is less than the rise of carbon emissions in per unit product, and total carbon emissions rise; when *ε* is large, the decrease in demand is greater than the rise of carbon emissions in per unit product, and total carbon emissions decrease.

Fig 7 shows the effect of *ε* on manufacturer and retailer’s profits. With *ε* rising, manufacturer revenues fall while retailer profits rise. The wholesale price of producer and the demand decrease, while the cost of technological upgrading increases for a same reduction amount per low-carbon product, leading to a decrease in producer’s revenue. The price of retail decreases, but the wholesale price decreases even more, making the difference between the retail and the wholesale prices increase, even though the market demand has a small drop, the price difference increases even greater, making the profit of retailer increase.

### 5.3. Effect of *λ* on equilibrium results

**Fig 8:**
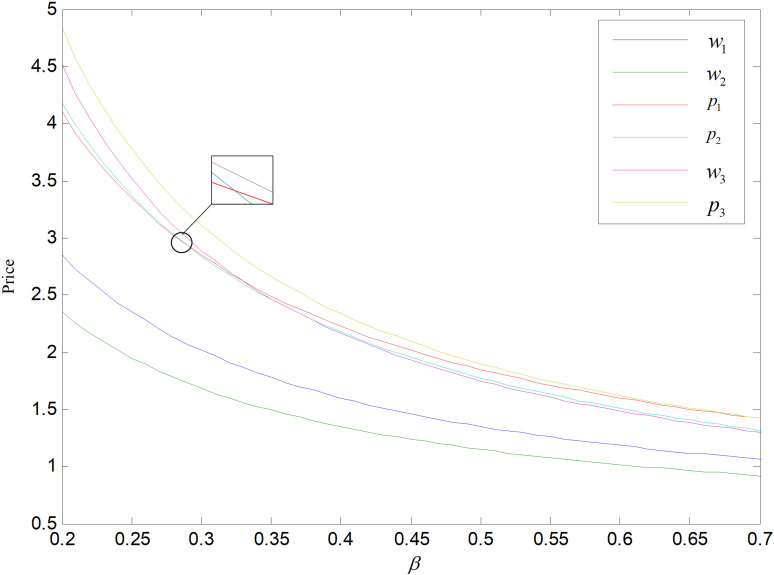
Effect of *β* on price.

**Fig 9:**
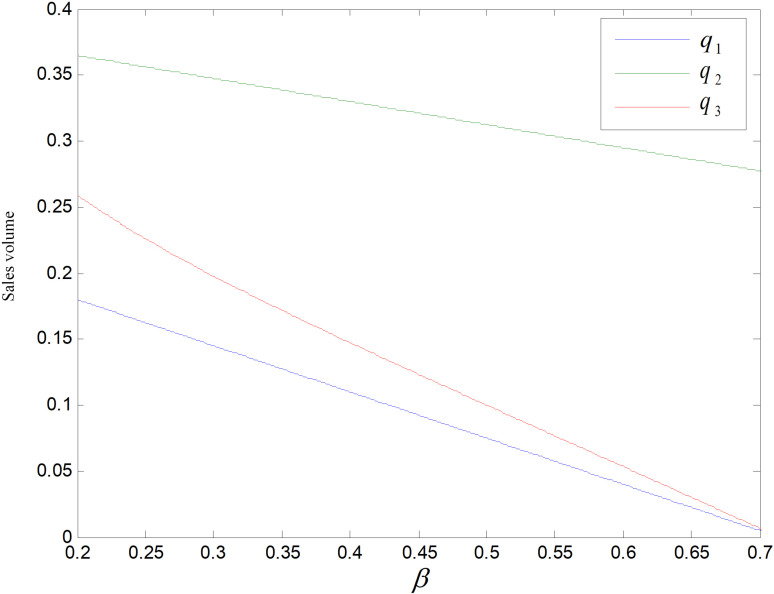
Effect of *β* on sales volume.

**Fig 10:**
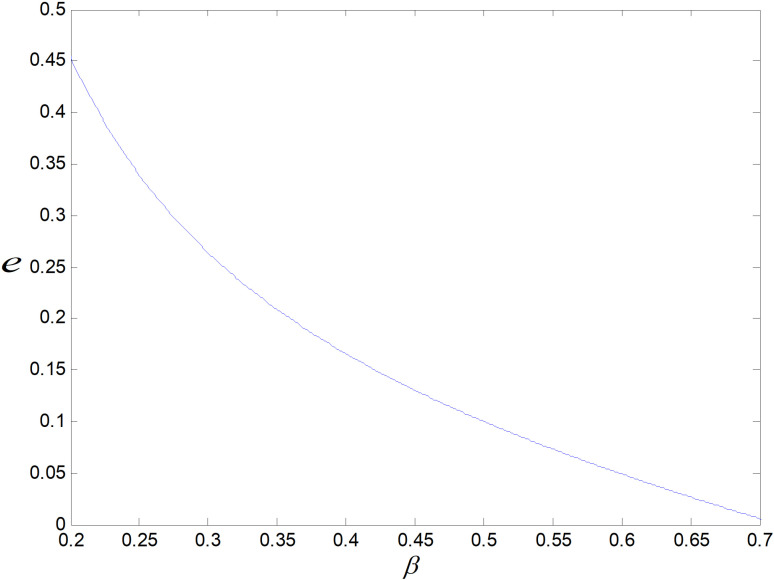
Effect of *β* on *e.*

**Fig 11:**
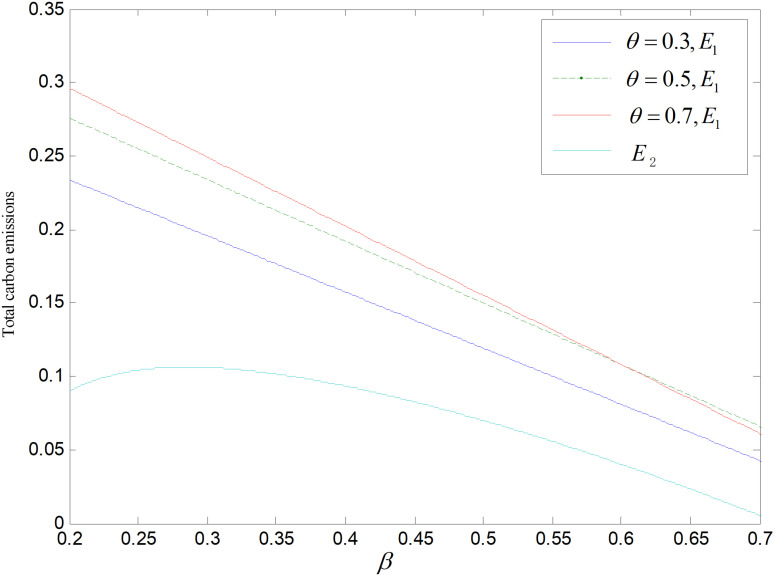
Effect of *β* on total carbon emissions ε=2.

**Fig 12:**
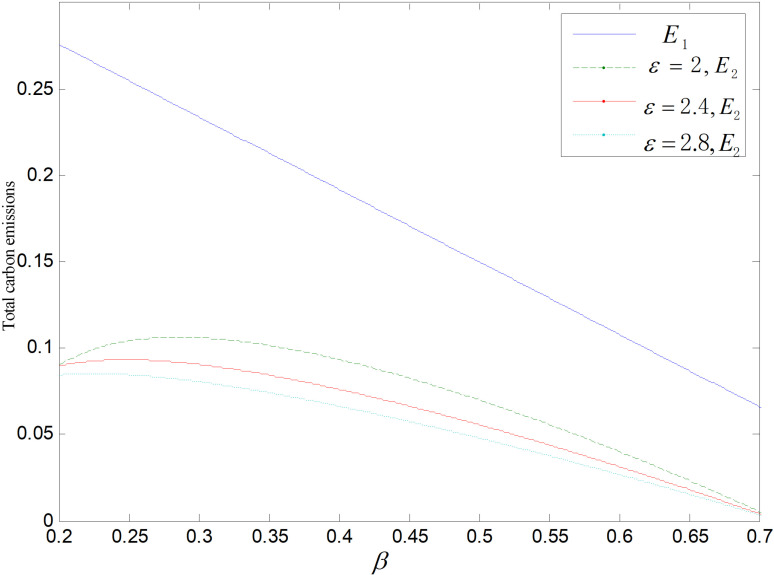
Effect of *β* on total carbon emissions (θ=0.5).

**Fig 13:**
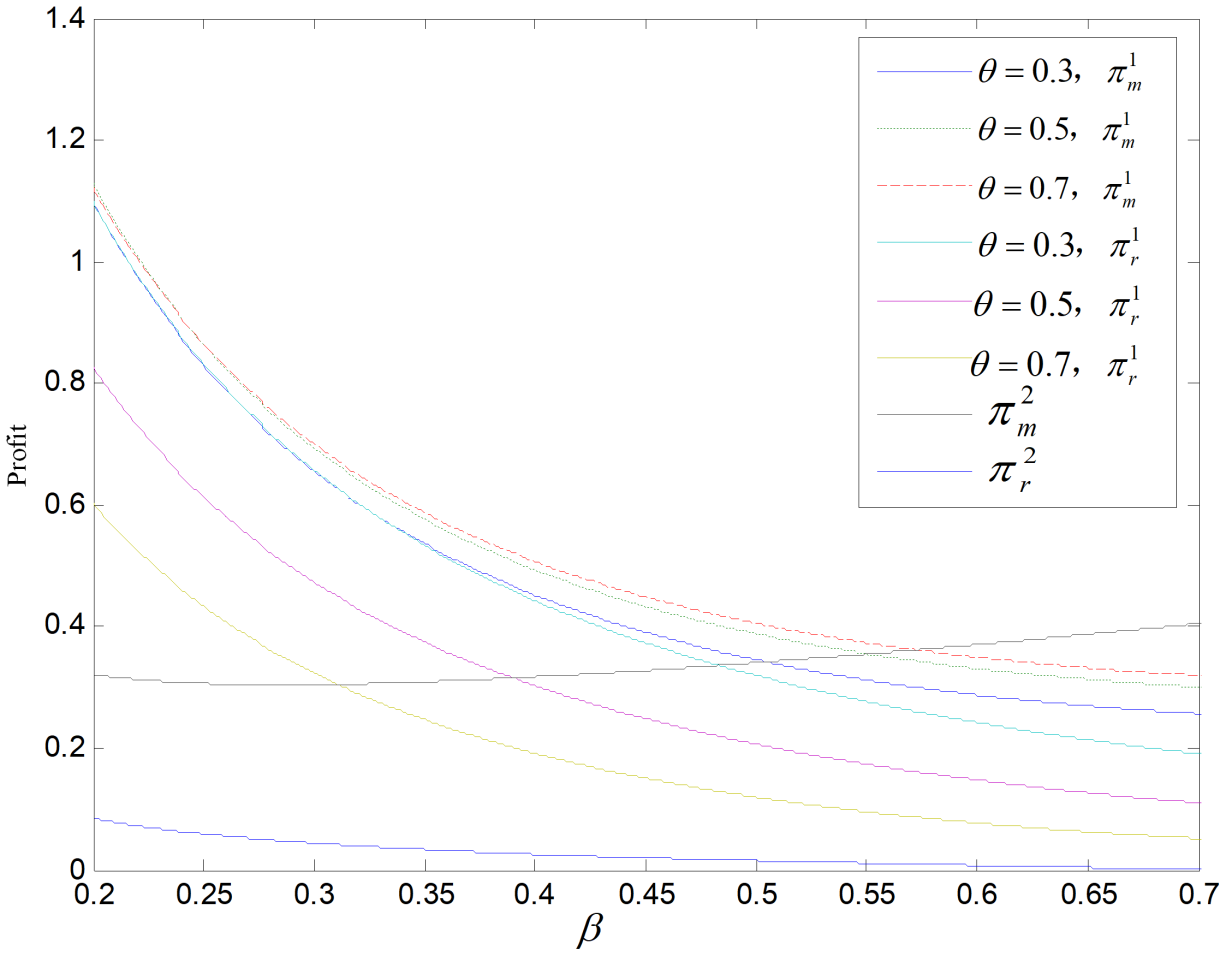
Effect of *β* on profit (ε=2).

**Fig 14:**
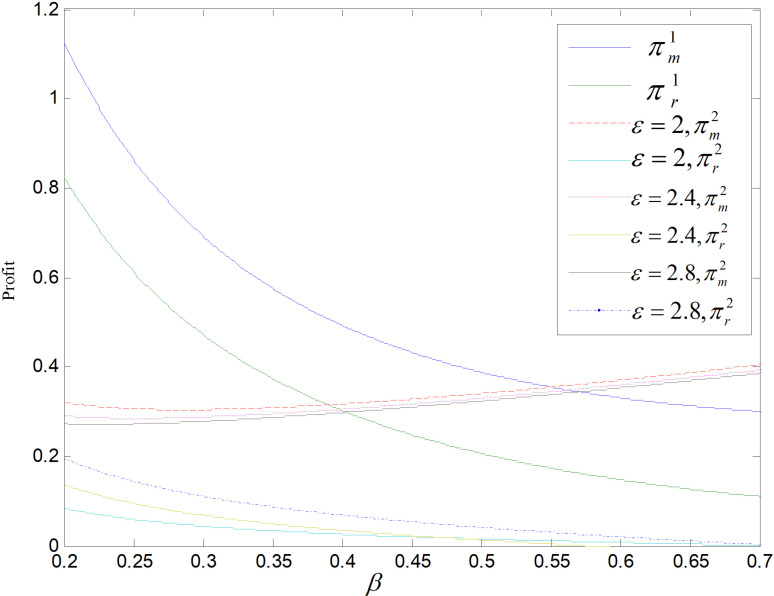
Effect of *β* on profit (θ=0.5).

According to the constraints in Chapter 4, the consumer price sensitivity coefficient $\beta \in \left[0.2,0.7\right]$ is obtained when the other parameters take the same values. When *β* is in this interval, its effects on price, sales volume, total carbon emissions and optimum carbon reduction per unit of the product are shown in Fig 8–14. Since the re-products are modified based on recycled products, their production cost is the lowest, While the cost of producing low-carbon goods is the same as that of ordinary goods, they also have a fixed cost associated with technological upgrading, which must be allocated to each low-carbon good, thus low-carbon goods cost the most. Producer determines the wholesale price based on cost inputs, so the wholesale prices of the three goods $w_{3}>w_{1}>w_{2}$. Thus $p_{3}$ is the highest retail price. When β is small, consumers are less price sensitive and price has little effect on consumers’ market demand, and are more likely to purchase reproductions than common products, so $p_{2}$ is larger than $p_{1}$. Consumers are more price sensitive when *β* increase, reproductions’ cost will be lower, and there is a large price reduction space. Manufacturers can spur an increase for the demand of reproduction by reducing price to boost enterprise profit.

Fig 9 shows the impact of *β* on demand, the demand of three products is negatively related to *β* and $q_{2}>q_{3}>q_{1}$. As *β* rises, consumers’ price sensitivity rises and their tendency to purchase decreases, and the demand for three items all decline, while reproductions’ demand decreases less. Reproducts have lower carbon emissions compared to common products, and lower prices compared to low-carbon goods, and consumers prefer cheap prices and low-carbon items, so the market demand for them is the highest.

Fig 10 shows that the consumer price sensitivity coefficient *β* and the optimal carbon emission reduction per low-carbon product are inversely connected. As *β* increases, the lower the consumer’s acceptance of the price of low-carbon goods, the lower the purchase intention will be. If consumer carbon reduction sensitivity is unchanged, the only method to boost the demand of low-carbon items is reducing their emissions more.

Figs 11 and 12 display the impact of *β* on overall carbon emissions under the two reduction strategies. The total emissions of the recycling remanufacturing plan are more than those of technology upgrading plan and fall down as *β* goes up. Emissions under technology upgrading strategy increase slightly before falling as *β* rises. The overall carbon emissions of the recycling and remanufacturing strategy is higher than that of the technology upgrading mode because common items and reproductions can be sold to two markets separately, and the market capacity is bigger than the other. And with the rise of *β*, consumers become less likely to buy goods, and the market demand of both common products and remanufactured products decreases, which makes their total carbon emissions decrease. And the optimal reduction of carbon emissions and demand for low-carbon items both decrease along with the rising of *β*, that is, the carbon output per unit of low-carbon items keeps rising and the need of low-carbon items keeps decreasing. When *β* is low, the increase of carbon output of low-carbon items is larger than the decrease of demand, which raises the overall carbon output. When *β* gets larger, overall amount of carbon emissions decreases because the increase of carbon output from low-carbon products is smaller than the decrease in demand.

The relationship between the retailer’s and manufacturer’s profits and *β* in these two modes are shown in Figs 13 and 14. The profits of retailers in the recycling mode are higher compared to those in the technology upgrade mode. As *β* increases, manufacturers’ profits in recycling and remanufacturing mode are firstly higher and then lower than those in the technology upgrading mode. That is, when *β* is small, the manufacturers’ profits are higher in recycling and remanufacturing mode than in technology upgrading mode. The sales of both products decrease and the profits of retailers and manufacturers decrease as *β* increases in recycling and remanufacturing mode. In contrast, in technology upgrading mode, the profit of manufacturer rises, while the profit of retailer falls as *β* increases. For *β* increases, the orders of low-carbons decrease, and retailer’s revenue is derived from the sale of low-carbons, so the retailer’s profit decreases. The producer’s profit comes from carbon allowances and the selling of low-carbon products. As *β* increases, the selling of low-carbon items decrease leads to a decline in total amount of emissions, and the firm will have a large number of carbon allowances available for sale, realizing the property of carbon allowances as a resource, and the profit rises.

### 5.4. Effect of *λ* on equilibrium results

**Fig 15:**
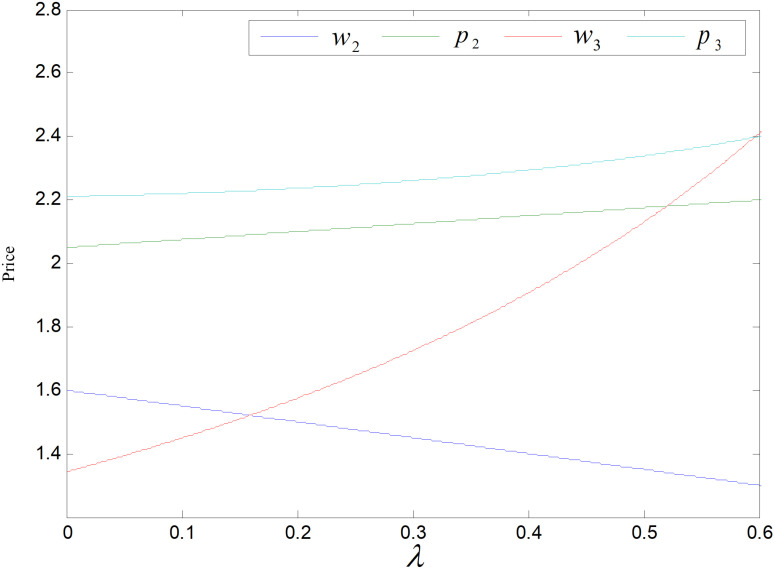
Effect of λ on price.

**Fig 16:**
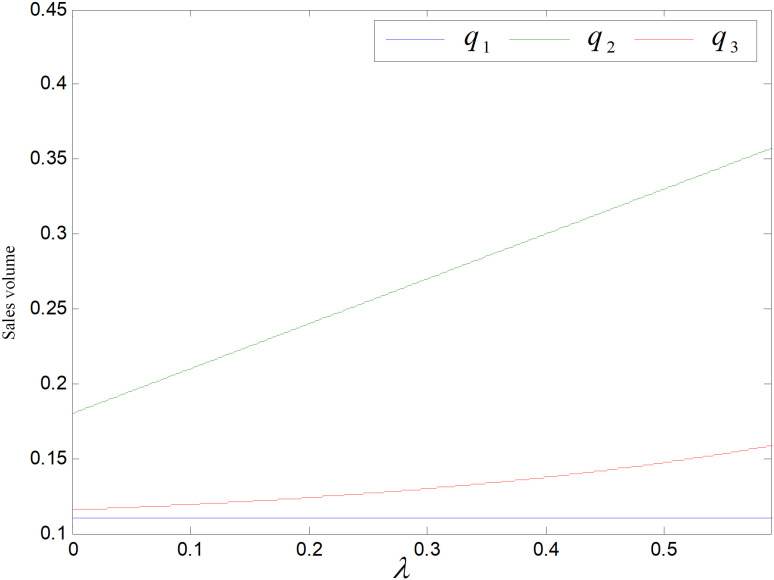
Effect of λ on sales volume.

**Fig 17:**
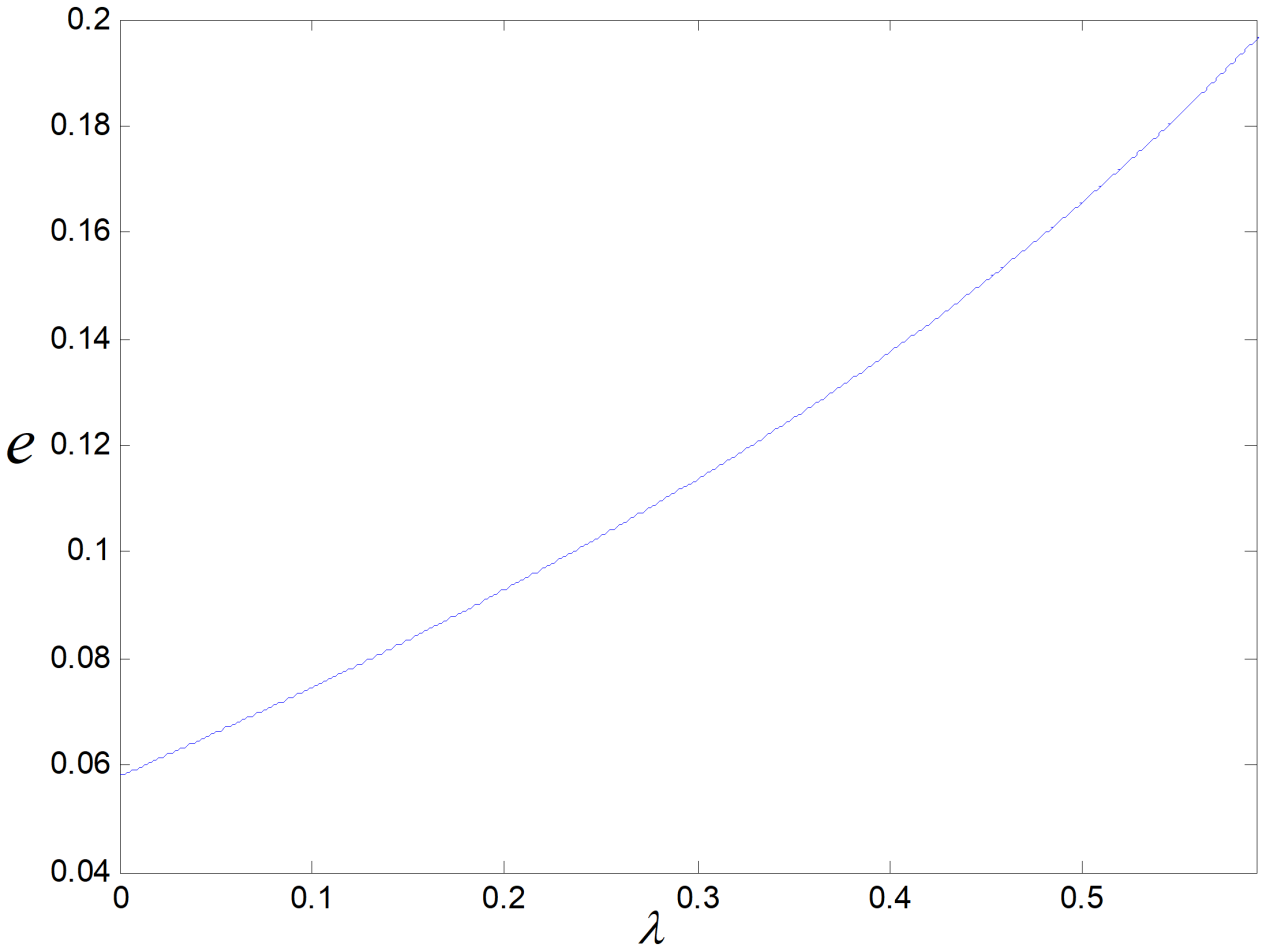
Effect of λ on e.

**Fig 18:**
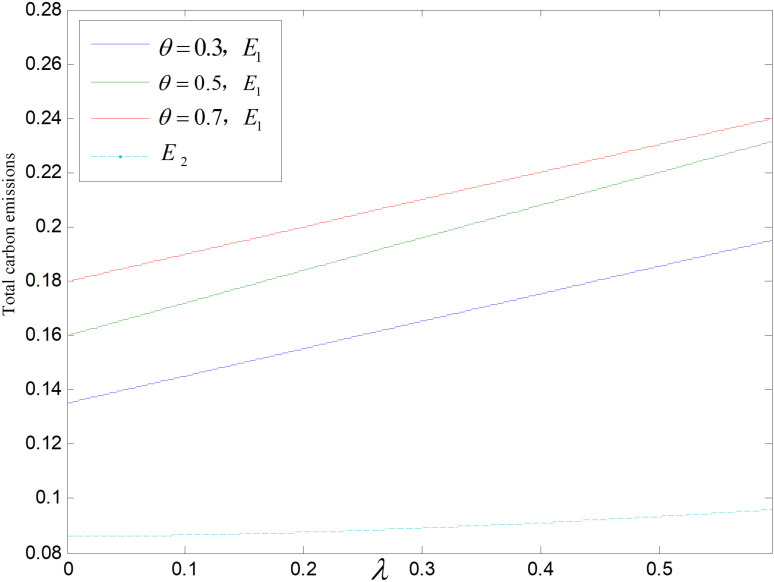
Effect of λ on total carbon emissions (ε=2).

**Fig 19:**
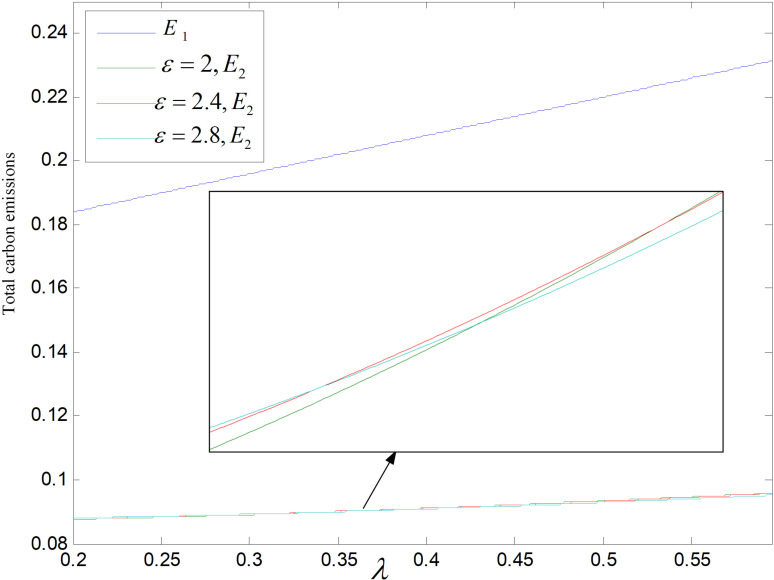
Effect of λ on total carbon emissionsθ=0.5.

**Fig 20:**
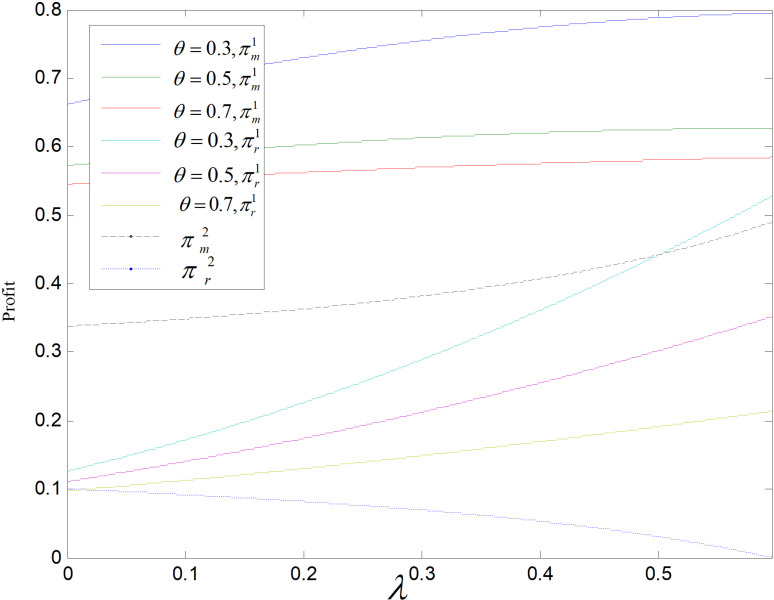
Effect of λ on profit (ε=2).

**Fig 21:**
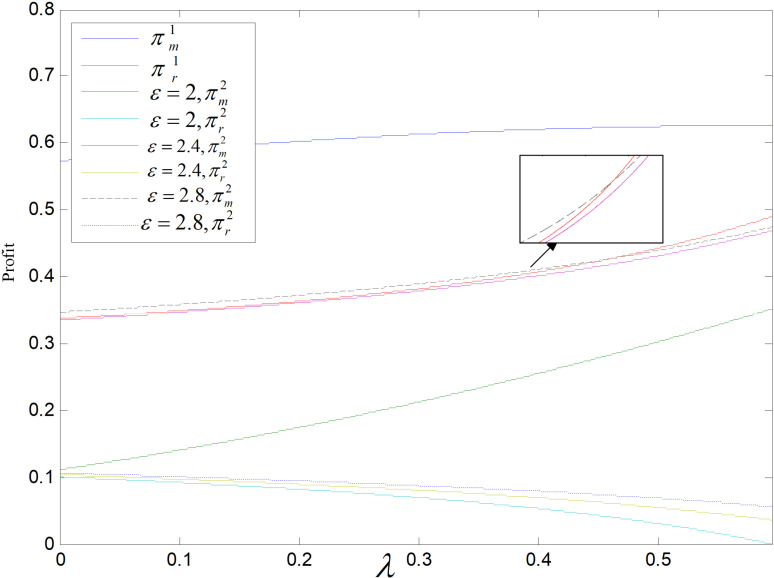
Effect of λ on profit (θ=0.5).

When the other parameters take constant values, the constraint function yields range of values of the emission reduction sensitivity coefficient, i.e., $\lambda \in \left[0,0.595\right]$. Figs 15–21 illustrates the effect of *λ* on optimal price, carbon emission reduction, market demand, total emission, and profit of each party in supply chain. Because common products do not reduce carbon emission and are sold to the markets different from the remanufactured products’, *λ* has no effect on the wholesale or retail prices of common products. Remanufactured goods’ wholesale price declines with the rising of *λ*, while its retail price rises. Both the retail and wholesale price of the low-carbon items increase while the retail’s increases more. When λ=0.595, the retail and wholesale price of the low-carbons are equal, as shown in Fig 15. Under the recycling and remanufacturing mode, consumers’ propensity to purchase remanufactured goods become stronger as *λ* increases, while the retail price and demand for remanufactured goods get higher. The retailers’ discourse power increases and they have greater bargaining power with the rising in sales volume, so the wholesale price of re-products declines. In technology upgrading mode, more consumers are inclined to buy low-carbons as λ increases, and the demand and the retail price become higher. At the same time, to cater to consumers’ increased carbon reduction sensitivity coefficient, manufacturers will increase the technology upgrading efforts, and the cost of technology upgrade needs to be apportioned to each product, so that the wholesale price of low-carbon items increases significantly until it equals to the retail price of low-carbons.

Fig 16 shows the effect of consumer carbon emission reduction sensitivity coefficient on demand. Common products’ demand is not affected by *λ*, and remanufactured and low-carbon items’ demands increase with *λ* increases. Compared to common products, re-products and low-carbon products can reduce carbon emission, and the larger *λ* is, the stronger the consumer’s propensity to purchase and increase more in demand with a fixed optimal carbon emission reduction of each product.

The optimal carbon emission reduction of per unit low-carbon item has a positive correlation with λ, as shown in Fig 17. Consumers will be more environmentally conscious as λ get larger, and their preference of low-carbon item get stronger, they will be more likely to purchase low-carbon items. To meet their demand, enterprises will increase technology upgrading efforts to reduce carbon emission of per product more effectively. At the same time, the demand for low-carbon items also rises with the decrease of carbon emission of per product, and the income of the enterprise will increase. As a result more funds can be used for technological upgrading, and the enterprise will enter a virtuous cycle of emission reduction and energy saving.

The effects of on total emλissions in both modes are shown in Fig 18,19. Similarly, carbon emissions are higher in recycling and remanufacturing mode than those in technology upgrading mode. As λ increases, consumers’ willingness to purchase both remanufactured and low-carbon products increase, and although the carbon emissions per unit of low-carbon products keep decreasing with the increase of λ, the decrease is smaller than the increase in market demand, which eventually leads to an increase of total carbon emissions.

Figs 20 and 21 show the effect of the consumer carbon reduction sensitivity coefficient on the supply chain participants’ profits. Manufacturers’ profits are positively related to λ in both modes, retailers’ profits are correlated positively with λ in the recycling and remanufacturing mode, and they are correlated negatively with λ in the technology upgrading mode. In the recycling-remanufacturing mode, as λ increases, the sales of reproduced items increase and both retailers and producers’ profits increase. In technological upgrading mode, as λ increases, the retail and wholesale price of low-carbons gradually become equal, then the profit of retailer becomes smaller and smaller until it will be zero at *λ* = 0.595. Both manufacturers and retailers are more profitable in the recycling and remanufacturing mode than in the technology upgrading mode.

### 5.5. Effect of $p_{c}$ on equilibrium results

**Fig 22:**
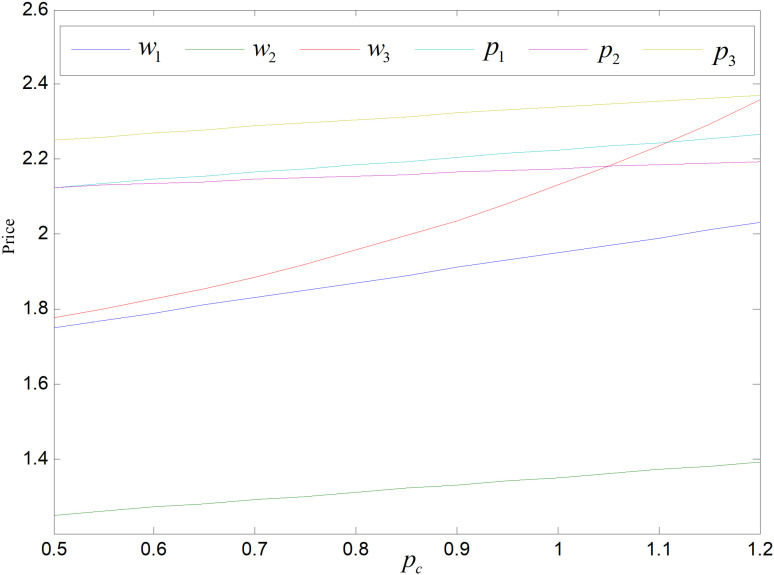
Effect of $p_{c}$ on price.

The price of carbon is an important factor to save energy and reduce emission through carbon trading policy. When the value of other factors remains unchanged, the range of carbon prices obtained according to the constraints, i.e., $p_{c}\in \left[0.5,1.22\right]$. Fig 22 shows the effect of carbon price on product pricing. Both retail and wholesale prices of the products in both modes climb in tandem with the price of carbon increase, and the wholesale price increase greater than the retail price. Manufacturers’ cost to purchase carbon quotas goes up due to the rise in carbon trading prices, spreading them to each product, and leading to all prices of the three items rising. Since the cost of carbon trading is borne by manufacturers, manufacturers will react more to the price rising of carbon, so the wholesale prices of these three items will rise more.

**Fig 23:**
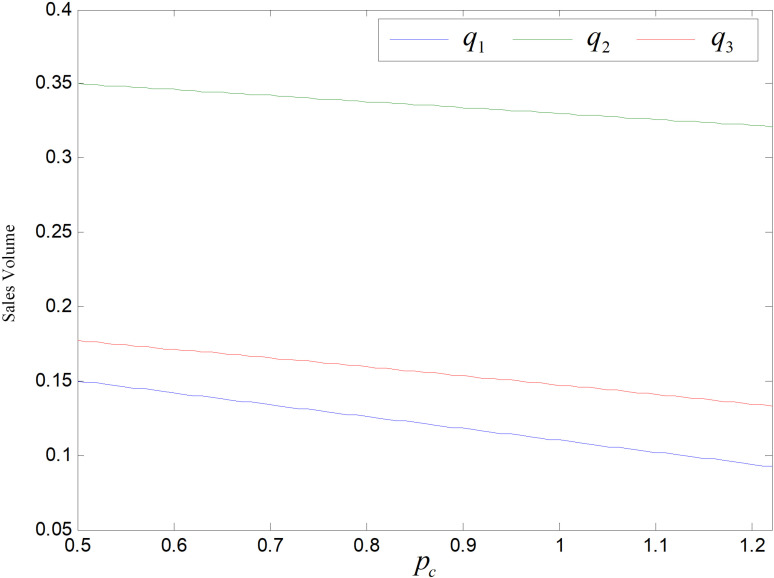
Effect of $p_{c}$ on sales volume.

The graph of carbon price’s effect on demand is shown in Fig 23. The prices of the three products all rises along with the increasing of carbon price, and the demands of the three goods all decrease for the purchaser are price sensitive.

**Fig 24:**
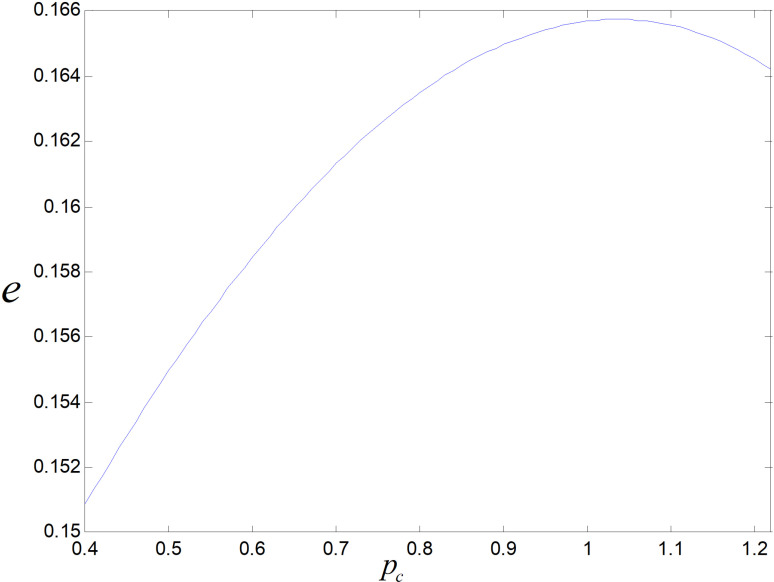
Effect of $p_{c}$ on e..

Fig 24 illustrates the reduction of unit low-carbon products keeps rising with the rising of carbon price. Due to the rising of carbon prices, enterprises will increase technological upgrading efforts to save carbon costs by lower the emission of each product.

**Fig 25:**
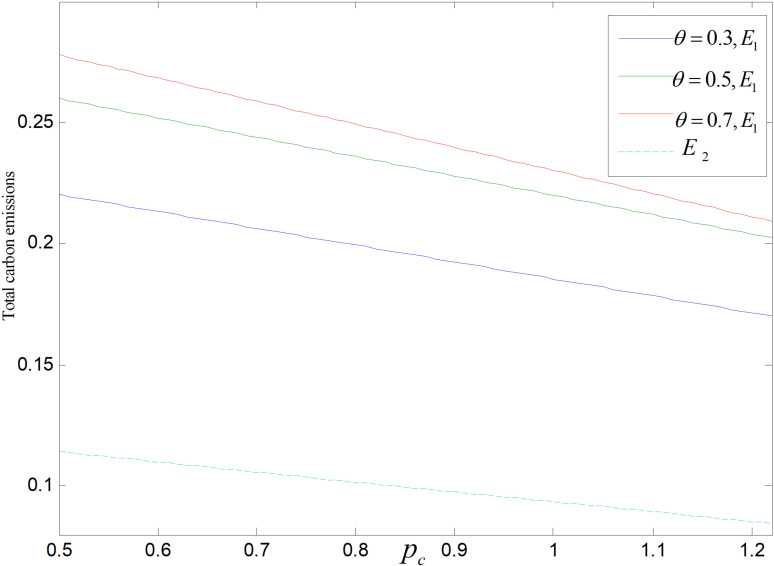
Effect of $p_{c}$ on total carbon emissions (ε=2).

**Fig 26:**
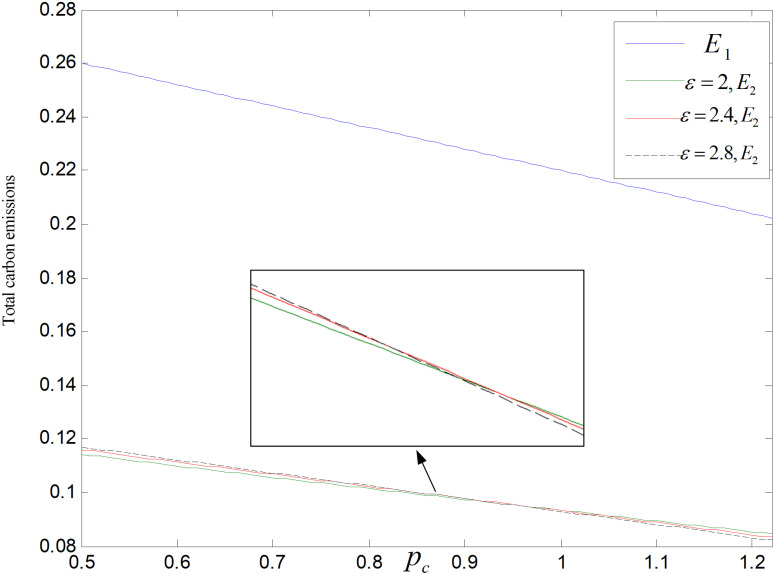
Effect of $p_{c}$ on total carbon emissions (θ=0.5).

Total carbon emissions are significantly impacted by the price of carbon. The total carbon outputs of both modes decrease with the rising of the carbon price, as shown in Figs 25 and 26. Prices of products will be raised by enterprises under the premise of ensuring profits, which leads to a decrease of consumer demand and total carbon emissions. In addition, in technology upgrading mode, enterprises will increase technological upgrading to lower carbon emission of per unit product, thus reducing the overall carbon outputs to a very low level.

**Fig 27:**
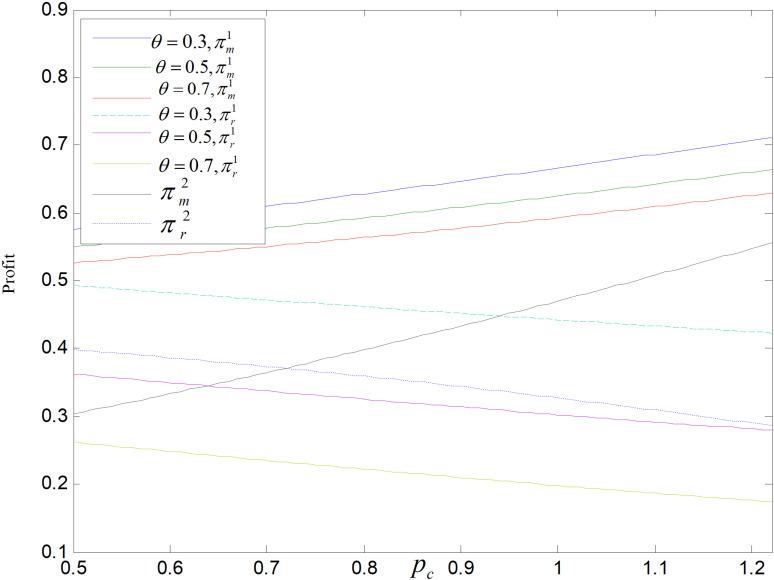
Effect of $p_{c}$ on total carbon emissions.

From Figs 27and28, we can find that in both modes, the manufacturer’s profit and carbon price are positively correlated, while the retailer’s is negatively correlated with it. The $p_{c}$ increases, the prices of three products all rise, although the market demand of all products declined, the manufacturer can earn income by selling carbon quotas, so the manufacturer’s profit increases. While retailers can only earn profit by selling products, the market demand decreases, and the gap between retail and wholesale price gently decreases, so the profit of retailers decreases. Manufacturers’ profits are higher in the recycling and remanufacturing mode than in the technology upgrading mode, but which mode is more profitable for retailers is uncertain and depends largely on $p_{c}$, *θ* and *ε*. When $p_{c}$ and *ε* are large, the retailer’s profit is larger in recycling and remanufacturing mode than in technology upgrading mode.

**Fig 28:**
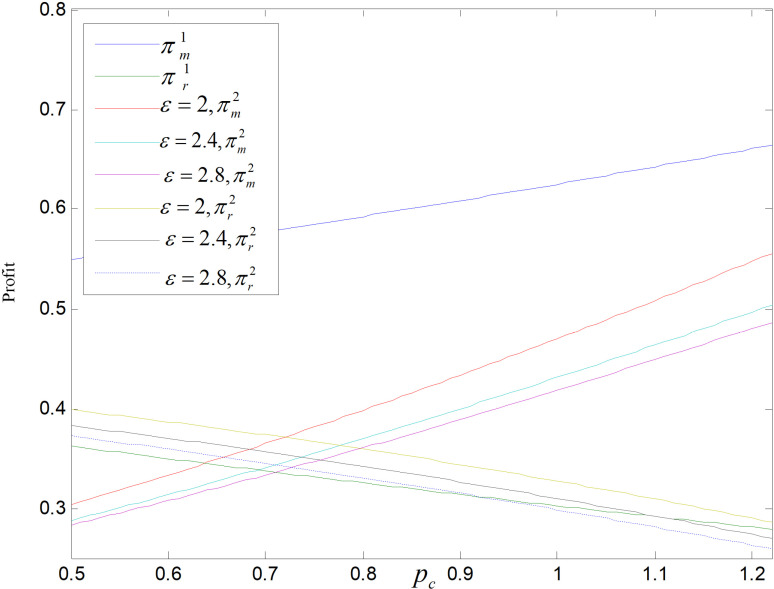
Effect of $p_{c}$ on profit.

From above simulation: it is found that technology upgrading requires the replacement of existing equipment, which is costly, resulting in the low-carbon products being more expensive than the re-products’, and the market demand is lower. The enterprise’s revenue under technology upgrading mode is low, but it can effectively reduce the total carbon outputs of the enterprise while remanufacturing can still decrease carbon emission of per unit product without increasing existing technological investment. The cost is low and the selling price is also low, which is widely liked by consumers, with higher market demand. But due to good sales, the enterprises’ total carbon outputs are higher. From the perspective of a rational economic person, when *β* is small, manufacturers will choose the most profitable way to conserve energy and decrease emissions through recycling and remanufacturing for the sake of corporate profit under the current cap-and-trade policy. When consumers are low price sensitive, low carbon preferences play an important role in their shopping. When *β* is large, carbon emissions of low-carbon products fall sharply and manufacturers can sell carbon allowances to make larger profits. At this point, manufacturers will choose to upgrade their technology. This is similar to the actual energy saving and emission reduction in real society. When *β* is large, most enterprises are willing to carry out recycling and remanufacturing but are unwilling to eliminate backward capacity through technological upgrading because of the high cost and low return of technological upgrading, and in the absence of government subsidies, enterprises lack the motivation to exchange old energy for new energy. When selling carbon allowances can bring profits to enterprises, they will choose to upgrade their technology. In both modes, increasing the price of carbon trading can increase manufacturers’ profits and also lessen total carbon outputs effectively.

## 6. Conclusions

This paper analyzes the reduction effects of two emission reduction methods, recycling and remanufacturing and technology upgrading, and economic benefits for manufacturers under the cap-and-trade mechanism, as well as the impacts of carbon price, carbon reduction sensitivity coefficient, consumer price sensitivity coefficient, carbon emission discount coefficient for remanufactured products, and investment cost coefficient on the optimal results, all supply chain parties’ profits and overall carbon emission. It is found that the total carbon emission is adversely connected to the price sensitivity coefficient and the carbon price in recycling and remanufacturing case. And the optimal carbon emission reduction of per unit low-carbon product is negatively correlated with technology upgrade coefficient, and positively correlated with the carbon sensitivity coefficient and the price of carbon in technology upgrading case. Finally, an arithmetic example was used to confirm the effects of influencing factors on these two carbon reduction methods, among which carbon price is the most efficient way to control carbon outputs. When consumer price sensitivity coefficient is low, due to the large investment in technology upgrading, manufacturers’ profits are not high, resulting in a manufacturer-led supply chain with little incentive to carry out technological innovation. So manufacturers will choose the recycling and remanufacturing approach to reduce emissions, but its total carbon emissions are higher than that of technology upgrading, and the reduction effect is poor. By constructing a Stackelberg game model of supply chain emission reduction, this paper deeply analyzes the impact of manufacturers ‘ recycling and remanufacturing and technological upgrading on supply chain carbon emissions and profits, and fills the gap that manufacturers sell multiple products or multiple product lines based on consumer heterogeneity under the carbon trading mechanism. It provides a new perspective and idea for the study of manufacturers’ independent emission reduction. Through sensitivity analysis, the influence of different parameters (such as consumer price sensitivity coefficient, consumer carbon emission reduction sensitivity coefficient, carbon trading price, emission reduction cost coefficient, etc.) on the decision-making results is discussed, which not only verifies the effectiveness of the carbon trading system, but also provides a strong support for the applicability of the theoretical model. Compared with the existing carbon emission reduction literatures, which mainly focuses on social welfare, this paper pays more attention to the actual carbon emissions of the two emission reduction methods. It is concluded that the emission reduction effect of recycling and remanufacturing is not as good as that of technology upgrading. This paper enriches the research content of the cross field of supply chain management and environmental sustainable development, especially provides more detailed and in-depth insights into the choice of carbon emission reduction strategies.

This paper provides a scientific decision-making basis for supply chain managers and policy makers. For managers from the enterprises, understanding the carbon emission reduction potential and economic feasibility of technology upgrading and recycling and remanufacturing can help them make optimal choices based on their own characteristics and market environment, achieving a win-win situation of economic and environmental benefits. Especially in the context of global climate change and increasing resource constraints, the circular economy and green supply chain management concepts emphasized in this article are of great significance for promoting industrial transformation and upgrading, enhancing international competitiveness, and accelerating the realization of China’s “double carbon” goals. This paper reveals the key role of carbon trading mechanism in supply chain carbon emission reduction decisions and provides a theoretical basis for the government to formulate more accurate and effective environmental policies. The “double carbon” target brings new opportunities and challenges to Chinese economy. As main force of carbon emission reduction, manufacturers should not only take immediate profits as the main basis for strategic decision-making, but also choose emission reduction methods with a strategic vision, seize transformation opportunities, invest moderately ahead of schedule, and promote technological upgrading. The future competition of enterprises mainly appears in the form of supply chain competition where they are located. Other supply chain member companies can share the cost of technological upgrading or improve the income of producers by cost-sharing or benefit-sharing contracts to reduce supply chain emission and boost the competitiveness of the whole supply chain. As the initiator of carbon trading policy, government’ main purpose is to save energy and lower enterprises’ carbon outputs. During the actual application phase, most enterprises adopt recycling and remanufacturing to lower emissions under the mechanism, so carbon emissions have decreased, but are still at a relatively high level. If the governments want the cap-and-trade policy to be more effective in reducing emissions, they should increase efforts in policy making (such as subsidies) to guide enterprises to technological upgrading, which can increase corporate profits while reducing emissions, and enterprises can realize green production without affecting profits. The government can also moderately increase the carbon trading price, promote enterprises to increase technological investment, improve the amount of carbon emission that are reduced by per unit of low-carbon products, reduce the overall carbon emissions, rise manufacturers’ profits, and motivate technological innovation. So the manufacturers enter a virtuous cycle of low carbon and high efficiency, change the mode of economic development, and effectively convert the economic benefits of enterprises into ecological benefits and social benefits.
